# The Role of Acupuncture in the Management of Insomnia as a Major or Residual Symptom Among Patients With Active or Previous Depression: A Systematic Review and Meta-Analysis

**DOI:** 10.3389/fpsyt.2022.863134

**Published:** 2022-04-15

**Authors:** Fei-Yi Zhao, Gerard A. Kennedy, Sarah J. Spencer, Russell Conduit, Wen-Jing Zhang, Qiang-Qiang Fu, Zhen Zheng

**Affiliations:** ^1^School of Health and Biomedical Sciences, RMIT University, Bundoora, VIC, Australia; ^2^Department of Nursing, School of International Medical Technology, Shanghai Sanda University, Shanghai, China; ^3^Shanghai Municipal Hospital of Traditional Chinese Medicine, Shanghai University of Traditional Chinese Medicine, Shanghai, China; ^4^School of Science, Psychology and Sport, Federation University, Mount Helen, VIC, Australia; ^5^Institute for Breathing and Sleep, Austin Health, Heidelberg, VIC, Australia; ^6^ARC Centre of Excellence for Nanoscale Biophotonics, RMIT University, Bundoora, VIC, Australia; ^7^Yangpu Hospital, School of Medicine, Tongji University, Shanghai, China

**Keywords:** acupuncture, depression, insomnia, systematic review, meta-analysis, RCT

## Abstract

**Background:**

Due to concerns about risks associated with antidepressants and/or hypnotics, complementary therapies such as acupuncture have been sought by patients with active or previous depression to manage insomnia. This systematic review aimed to clarify if acupuncture is effective and safe enough to be recommended as an alternative or adjuvant therapy to standard care in ameliorating concomitant or residual insomnia, two types of insomnia associated with depression.

**Methods:**

Randomized controlled trials (RCTs) of depression-related insomnia (DI) treatment *via* acupuncture *vs*. waitlist-control or placebo-/sham-acupuncture and RCTs of DI treatment *via* acupuncture alone or combined with standard care [Western pharmacotherapy and/or cognitive-behavioral therapy (CBT)] *vs*. standard care alone were searched for from seven databases from inception to December 2021. Cochrane criteria were followed.

**Results:**

Twenty-one studies involving 1,571 participants were analyzed. For insomnia as a major symptom of active depression, meta-analyses suggested that acupuncture significantly reduced the global scores of both the Pittsburg Sleep Quality Index (PSQI) [MD = −3.12, 95% CI (−5.16, −1.08), *p* < 0.01] and Hamilton Depression Scale (HAMD) [SMD = −2.67, 95% CI (−3.51, −1.84), *p* < 0.01], in comparison with placebo-acupuncture. When compared with conventional pharmacotherapy (antidepressants and/or hypnotics), the results favored acupuncture in decreasing PSQI [MD = −1.17, 95% CI (−2.26, −0.08), *p* = 0.03] and HAMD [SMD = −0.47, 95% CI (−0.91, −0.02), *p* = 0.04]. Acupuncture was comparable to conventional pharmacotherapy in reducing scores of each domain of PSQI. For insomnia as a residual symptom of previous or partially remitted depression, acupuncture conferred a very limited, non-significant therapeutic advantage against sham-/placebo-acupuncture. Whether acupuncture has an add-on effect to conventional pharmacotherapy in this type of insomnia has not been investigated. Also, no study was available to address the efficacy differences between acupuncture and CBT or the synergistic effect of these two therapies.

**Conclusions:**

There is a low to moderate level of evidence supporting acupuncture as a safe and effective remedy alternative to or adjuvant to conventional pharmacotherapy (antidepressant and/or hypnotic) in improving insomnia and other depression symptoms among patients with active depression. Furthermore, the patients' complaint of disrupted sleep continuity is most likely to benefit from acupuncture. The benefit of acupuncture on residual insomnia associated with previous or partially remitted depression is limited. Future acupuncture studies need to consider applying optimal dosage and addressing deficiencies in trial quality.

**Systematic Review Registration:** https://www.crd.york.ac.uk/prospero/display_record.php?ID=CRD42021269880, PROSPERO, identifier: CRD42021269880.

## Background

Depression is an increasing global burden and a common mental illness that affects both the psychological and physiological health (1), which in turn can severely limit social and cognitive functioning and diminish quality of life (2, 3). More than 300 million individuals throughout the world are estimated to suffer from depression, which is ranked by World Health Organization as the single largest contributor to global disability (1, 4). Disturbed sleep is the most prominent symptom among patients with depression (5). Up to 67–84% adults and 57% children and adolescents experience insomnia during depressive episodes (6). The polysomnography (PSG) data of depressed patients also reveal that they generally have extended sleep-onset latency (SOL), decreased sleep efficiency (SE), frequent nocturnal awakenings, early morning awakening, and reduced slow-wave sleep (SWS) (7, 8). Worst of all, sleep disturbances are usually underestimated or overlooked and are seldom a treatment target, given the common assumption that sleep problems are a concomitant symptom of depression and will diminish as depression subsides (5). This is despite evidence that depressive symptoms in some patients will not improve until sleep issues are resolved (9). Notably, insomnia is not only linked to more severe depressive symptoms, including suicidality, but is also associated with a poorer response to treatments for depression (6, 8), a longer duration of treatment (5), a slower recovery from their illness (2), and a lower rate of remission (5). In addition, persistent insomnia is usually a residual symptom of depression (10), occurring in approximately 95% of patients with incomplete remission of depression and 72% patients who have full remission following antidepressant treatment (6). These findings indicate that isolated antidepressive therapy may not provide an adequate remedy for patients with sleep problems (6). Consequently, resolving insomnia as a major or residual symptom in patients with active or previous depression, respectively, is crucial and has significant clinical relevance, because the former can interact with depression and thereupon then exacerbates and maintains each condition (11) and the latter constitutes a pivotal risk factor for a future depressive episode (relapse into depression) (6, 12) and contributes to undesirable clinical outcomes (5).

Cognitive-behavioral therapy for insomnia (CBT-i) is recommended as the most efficacious non-pharmacological therapy for insomnia and has also shown a therapeutic effect for DI (5, 13). Unfortunately, access to CBT-i treatment is restricted due to cost and a dearth of trained CBT-i providers, particularly in community-based sleep clinics and rural areas (14). Poor adherence is another challenge in delivering CBT-i in patients with DI, as insomniacs with elevated depressive symptom are more likely to terminate and drop out from CBT-i treatment early (15). The preferred class of drugs for DI is sedating antidepressant agents, including trazodone and mirtazapine (7, 8) or agomelatine (a melatonergic agonist and a 5-HT_2c_ antagonist) (16). Despite satisfactory curative effects, dependence and complaints such as dry mouth, nausea, dizziness, increased appetite and weight gain, and/or constipation (16, 17) reported during the course of treatment can result in treatment termination in some patients (18).

Complementary and alternative medicine (CAM) is becoming popular in the management of the insomnia–depression–anxiety symptom cluster (19); some evidence-based clinical practice guidelines (CPGs) in the Euro-American and Asian-Pacific regions include CAM therapies to inform clinicians' practice decisions on various types of insomnia (20). Acupuncture, an integral component of CAM, is a non-pharmaceutical therapy of traditional Chinese medicine (TCM) that stems from ancient clinical practice and has been widely practiced in China for over 4,000 years (21, 22). It involves the insertion of thin, sterile needles into the skin at defined sites (called “acupoints”) for therapeutic purposes (21, 23, 24). After insertion, the needles are usually twisted back and forth manually (manual acupuncture, MA) or connected to an electric microcurrent device delivering either high- or low-frequency impulses (electroacupuncture, EA), or a combination of both techniques is used (22, 24).

Acupuncture has been extensively utilized in clinical practice for the management of DI, and many randomized Controlled trials (RCTs) have been published (25). Nevertheless, the inconsistent findings (26, 27) and vast differences between research designs (25) disallow definitive conclusions with respect to the recommendation of acupuncture for DI. In light of the challenges in the current management of DI, we conducted this systematic review, aiming to address the following research questions: (1) could acupuncture be used as an isolated remedy for DI; (2) how effective and safe was acupuncture in the treatment of DI in comparison with standard care; and (3) when acupuncture was applied as an adjunctive remedy of standard care, could it further enhance the efficacy or minimize the side effects of Western pharmacotherapy? The current systematic review was carried out in accordance with the Cochrane Handbook for Systematic Reviews (28) and was reported following the *Preferred Reporting Items for Systematic Reviews and Meta-Analyses (PRISMA) 2020 Statement* guidelines (29).

## Materials and Methods

### Study Registration

The protocol for this systematic review was registered in the Prospective Register of Systematic Reviews (PROSPERO): no. CRD42021269880.

### Eligibility Criteria

Studies included were formally published RCTs with parallel designs. Regardless of gender, race, and age, patients with a clinical diagnosis of depression as per standard diagnostic criteria and with insomnia as the major or residual symptom were included. Any trial without a standard diagnostic guideline for depression [e.g., *Diagnostic and Statistical Manual of Mental Disorders*, fourth edition (DSM-IV); *Diagnostic and Statistical Manual of Mental Disorders*, fifth edition (DSM-V); *International Classification of Diseases*, 10th edition (ICD-10); *International Classification of Diseases and Related Health Problems*, 11th edition (ICD-11); and *Chinese Classification of Mental Disorders*, third edition, (CCMD-3)] was excluded. Distinguished from general depression, depression associated with specific physiological stages in women (e.g., antenatal depression, postpartum depression, and perimenopausal depression) possesses a unique etiology and pathogenesis. Studies examining insomnia related to these specific depressions were not included in this review. Patients were excluded if poor sleep was caused by a sleep disorder(s) other than insomnia, or they had other sleep disorder(s) in addition to insomnia. Studies involving patients with comorbid DI and cardiovascular disease, cancer, cerebrovascular disease, endocrine diseases, other psychiatric and mental disorders (e.g., anxiety and schizophrenia), or other severe disorders were also excluded. There was no restriction based on the severity of either depression or insomnia. Interventions were restricted to the traditional needle acupuncture (TNA) including MA and EA or TNA combined with standard care for DI (antidepressants and/or hypnotics and sedatives and/or CBT-i). Comparator interventions were restricted to waitlist-control, placebo-/sham-acupuncture, or standard care. It should be explained that sham-acupuncture refers to a needle superficially placed in a region close to but not an acupoint (30); placebo-acupuncture such as Streitberger needles (31) or other blunt needle/device refers to a needle placed on the skin surface (not penetrating the skin) at the same acupoint as that of verum acupuncture or at a region beside an acupoint (30). The primary outcome was self-reported, validated sleep scales/questionnaires [e.g., Pittsburg Sleep Quality Index (PSQI), Insomnia Severity Index (ISI), and Athens Insomnia Scale (AIS)] and/or objective sleep parameters measured by sleep monitoring devices. Secondary outcomes included total clinical effectiveness rate, adverse events, and/or depression scales/questionnaires [e.g., Hamilton Depression Scale (HAMD) and Self-rating Depression Scale (SDS)]. There are several versions of HAMD, such as HAMD-6, HAMD-17, HAMD-21, HAMD-23, HAMD-24, and HAMD-27 (32). We did not include a restriction on the HAMD version for searching and including studies. Papers were excluded if they did not report the global scores of any validated sleep scale/questionnaire, even though they reported the clinical effectiveness rates based on the scale or reported partial items/domains of the scale/questionnaire.

### Search Strategy and Data Extraction

Together with a professional medical librarian with a TCM background, we used filters to reliably identify studies and undertook a comprehensive search of three English electronic databases and four Chinese databases—MEDLINE (*via* PubMed), Cochrane Central Register of Controlled Trials (CENTRAL), and EMBASE and Chongqing VIP database (CQVIP), China Biomedical Literature Service System (SinoMed), Wanfang Database, and China National Knowledge Infrastructure (CNKI)—from their inception date until December 2021, without language restriction. The search was carried out by combining search terms from four categories: (1) acupuncture, (2) depression, (3) insomnia, and (4) RCT. Searches were supplemented by retrieval of other sources, including the online trial registries such as the US ClinicalTrials.gov, WHO International clinical trials registry platform search portal, and any additional articles meeting eligibility criteria that were cited in reference lists of the included papers and existing systematic reviews, to avoid potential omission (see Appendix 1 for detailed search terms and search strategies).

After screening the titles and abstracts, full texts were acquired and cross-checked for eligibility by two researchers (W-JZ and F-YZ). A predetermined data form was employed to extract the following information (demographic and clinical characteristics) from each study: identification information, publication year, types of DI (insomnia as a major symptom or a residual symptom of depression), grouping methods as well as number and gender of participants in each group, duration of DI, diagnostic criteria employed, TCM syndrome pattern of DI included, intervention protocols including acupuncture frequency, session and duration as well as acupoint selection, prescription in comparator (frequency, session, and duration in placebo-/sham-acupuncture; or type, dosage, and oral frequency of Western medication; or regimen of CBT-i), outcome measures, results, follow-up duration and results, and adverse events (AEs). We also tried to contact the corresponding author of the original RCT to access missing data or to clarify other unclear or uncertain information.

### Study Quality and Risk-of-Bias Assessment

Two evaluators (Q-QF and F-YZ) carried out standalone appraisal (including determining risk of bias and assessing the internal validity) of all the included studies using the Cochrane Collaboration's risk-of-bias tool (28). They also independently graded the methodological quality of each RCT on the basis of the modified Jadad scale ranging from 0 to 5 points [the scoring criteria and approach of the modified Jadad system refer to this review (33)]. The details of acupuncture procedure including completeness and reporting quality in each trial were described and appraised *via* the revised Standards for Reporting Interventions in Clinical Trials of Acupuncture (STRICTA) checklist (Version 2010) (34). If consensus could not be reached, a third assessor (ZZ) was consulted in resolving any discrepancies.

## Data Analysis and Evidence Quality Assessment

### Data Analysis

Available data were merged for quantitative meta-analysis *via* the Cochrane Collaboration Review Manager Software (RevMan Version 5.4.1). The inverse-variance approach in RevMan was applied to assign weight to each included trial. Given that the primary outcome measure, global scores of sleep scales/questionnaires or objective sleep parameters, was a continuous variable, mean differences (MD) with 95% confidence intervals (CI) between the intervention and control group were calculated. When sufficient data were available (number of RCTs ≥ 3), all domains of PSQI except for “Use of sleeping medication” were included for data synthesis. This item was not analyzed because in most RCTs included, drugs were not allowed in treatment groups adopting acupuncture. For individual RCTs with continuous outcomes measured by a variety of scales or different versions of the same scale (e.g., 6-item HAMD, 17-item HAMD, and 24-item HAMD), standardized mean differences (SMD) were adopted as recommended by Cochrane. Risk ratios (RRs) with 95% CIs were adopted for dichotomous data, such as the total clinical effectiveness rate. The level of heterogeneity across studies was tested using the *Q*-test and *I*^2^-test. Statistical significance was set at a two-tailed probability (*p*) < 0.05. We employed a fixed-effects model to pool data when the *p* > 0.10 in the *Q*-test and the *I*^2^ ≤ 50%, which was considered to be an acceptable level of heterogeneity. Otherwise, a random-effects model was adopted to provide a more conservative estimate of effect. For significant clinical heterogeneity, subgroup analyses were performed based on different acupuncture stimulations (MA or EA), principle of acupuncture prescription (fixed, semi-standardized, and individualized), treatment frequency [<5 sessions per week or ≥5 sessions per week; note that when the treatment frequency varied over the course of treatment, the frequency was calculated with reference to a previous high-quality systematic review (35)], needle retention time (<30 or ≥30 min), different therapeutic schedules of standard care (pharmacotherapy or CBT-i), different medication in the controls (antidepressant, hypnotic, or antidepressant + hypnotic), and different versions of HAMD used. With STATA software (Version 17.0), sensitivity analysis and meta-regression analysis were employed to explore sources of heterogeneity as well and check robustness of the conclusions. We also investigated publication bias where at least 10 trials were included in the meta-analysis by implementing Egger's test and Begg's test with STATA software (Version 17.0).

### Evidence Quality Assessment

The evidence quality, referring to the strength or reliability of study findings, was assessed by two independent raters (Q-QF and F-YZ), adhering to the Grades of Recommendation, Assessment, Development, and Evaluation (GRADE) framework, which classifies the certainty of evidence into four levels (36). The starting point for certainty in given effect estimates for RCTs is “High” but could be rated down as “Moderate,” “Low,” or “Very low” due to any restrictions with respect to risk of bias (that is, a risk of bias in the original RCTs), imprecision, inconsistency, indirectness, and publication bias (36).

## Result Analysis

**Figure 1:**
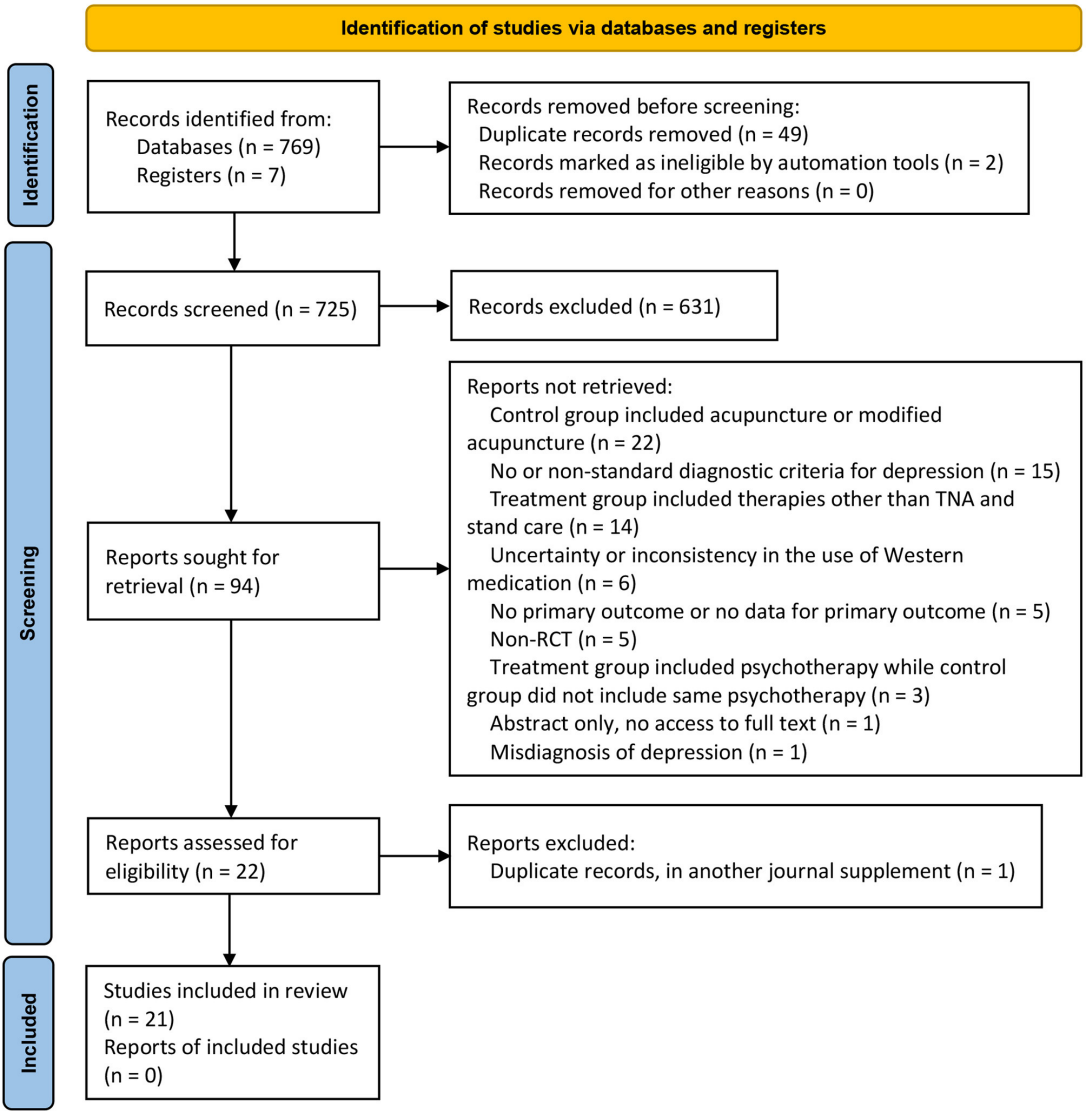
Flow diagram of the study selection process.

In total, 776 potentially relevant articles were identified based on our search strategy in the initial search. After the duplicates were removed and a careful full-text screening was done, 21 studies (involving 1,571 participants) met the predefined criteria (Figure 1). All included studies were qualitatively analyzed, and 19 of them underwent quantitative synthesis (meta-analysis). The discarded studies with detailed reasons of irrelevance are summarized in Appendix 2.

### Description of Studies

Of the 21 trials, two trials (26, 37) investigated the efficacy of acupuncture for depression-associated residual insomnia using a three-arm design with both placebo-acupuncture and sham-acupuncture as controls. The remaining 19 trials investigated the efficacy of acupuncture for active depression accompanying or comorbid with insomnia. Among them, three studies (27, 38, 39) employed placebo-acupuncture as control [study (27) was a three-arm RCT with both sham- and placebo-acupuncture as controls], 10 studies (40–49) employed standard care (antidepressant and/or hypnotics) as control, and the remaining six studies (50–55) compared the standard care (antidepressant and/or hypnotics) alone with acupuncture in addition to standard care. None of the 21 RCTs included CBT-i or waitlist-control. In the trials with pharmacotherapy as control, one trial used a hypnotic (clonazepam) (46) only, two trials used combined antidepressant and hypnotic [paroxetine + estazolam (40) and fluoxetine + eszopiclone (45)], and the rest used antidepressants only. The frequency of use of antidepressive or hypnotic agents from high to low were paroxetine (5/18), mirtazapine (4/18), venlafaxine (2/18), citalopram (1/18), escitalopram (1/18), sertraline (1/18), fluoxetine (1/18), clonazepam (1/18), estazolam (1/18), and eszopiclone (1/18). Eight out of the 21 trials (26, 27, 37, 40, 41, 44, 52, 54) appraised the clinical efficacy of EA, while the remaining 13 trials studied MA (Table 1).

**Table 1:** Study characteristics of 21 included studies.

**References**	**Group/size (M, male; F, female)**	**Age (year)**	**Depression duration (m, month; y, year)**	**Diagnostic system**	**TCM syndrome type**	**Acupuncture interventions**	**Acupoints selection**		**Prescription in control group (placebo or Western medication)**	**Outcome measure tool**	**Acupuncture/Acupuncture + Western medication compared with control (waitlist, placebo-/sham-acupuncture, Western medication)**	**Follow-up**	**Adverse events (AEs)**
							**Variation in acupoints**	**Acupoints**					
Yin et al. (27)▴	EA/*n* = 30 (11 M, 19 F) Sham-EA/*n* = 30 (10 M, 20 F) Placebo-EA/*n* = 30 (11 M, 19 F)	EA/47.30 ± 14.89 Sham-EA/49.80 ± 15.13 Placebo-EA/46.77 ± 15.57	EA/5.67 ± 5.70 y Sham-EA/7.48 ± 6.23 y Placebo-EA/5.89 ± 5.64 y	DSM-IV	NR	30 min/session, 3 sessions/week (once every other day) for 8 weeks (continuous wave, 30 Hz, 0.1–1mA)	Fixed	EX, EX-HN3, GV20, GV24, HT7, PC6, SP6	Sham-EA (superficial acupuncture) or Placebo-EA (Streitberger acupuncture) on non-acupoins, 30 min/session, 3 sessions/week (once every other day) for 8 weeks	(i) PSQI (ii) HAMD_17_ (iii) SDS (iv) HAMA (v) Actigraphy (SE, TST, ATs)	(i–i) compared with sham-EA *p* < 0.01 (i–ii) compared with placebo-EA *p* < 0.05 (ii–i) compared with sham-EA *p* < 0.01 (ii–ii) compared with placebo-EA *p* < 0.01 (iii–i) compared with sham-EA *p* < 0.01 (iii–ii) compared with placebo-EA *p* < 0.01 (iv–i) compared with sham-EA *p* < 0.01 (iv–ii) compared with placebo-EA *p* < 0.01 (v–i) TST, compared with sham-EA *p* < 0.05; SE, compared with sham–EA *p* < 0.05; ATs, compared with sham-EA *p* > 0.05 (v–ii) TST, compared with placebo-EA *p* < 0.01; SE, compared with placebo-EA *p* < 0.01; ATs, compared with placebo-EA *p* > 0.05	(i–iv) lower PSQI, HAMD, SDS, HAMA in EA at 4-week follow-up (v–i) no significant difference in TST, SE and ATs at 4-week follow-up between EA and sham-EA (v–ii) longer TST, higher SE in EA than in placebo-EA at 4-week follow-up; no significant difference in ATs at 4-week follow-up between EA and placebo-EA	EA/*n* = 3 [hand numbness and pain at acupoints] Sham-EA/*n* = 2 [hematoma (1), dizziness (1)] Placebo-EA/*n* = 1 [dizziness]
Qin et al. (38)▴	MA/*n* = 60 (23 M, 37 F) Placebo-MA/*n* = 60 (26 M, 34 F)	MA/39 ± 14 Placebo-MA/40 ± 14	NR	DSM-V	NR	30 min/session, 3 - 4 sessions/week (once every other day) for 4 weeks (Fluoxetine + Deanxit as basic treatment)	Fixed	BL18, EX, EX-HN1, EX-HN3, HT7, GV20, KI6, SP6	Placebo-MA (Streitberger acupuncture) 30 min/session, 3-4 sessions/week (once every other day) for 4 weeks at same acupoints (Fluoxetine + Deanxit as basic treatment)	(i) PSQI (ii) HAMD_24_ (iii) total clinical effectiveness rate	(i) Compared with placebo-MA *p* < 0.05 (ii) Compared with placebo-MA *p* < 0.05 (iii) Compared with placebo-MA *p* < 0.05	No follow-up	NR
Zhao et al. (39)▴	MA/*n* = 34 (11 M, 23 F) Placebo-MA/*n* = 33 (13 M, 20 F)	MA/43.87 ± 10.51 Placebo-MA/45.16 ± 11.39	MA/4.66 ± 1.35 m placebo-MA/4.01 ± 1.50 m	DSM-V, ICD-10	NR	30 min/session, 3 sessions/week for 8 weeks	Fixed	EX-HN1, GB13, GV11, GV24, HT7	Placebo-MA (Streitberger acupuncture) 30 min/session, 3 sessions/week for 8 weeks at same acupoints	(i) PSQI (ii) HAMD_17_ (iii) PSG (TST, SE, WASO, SOL, ATs, REM-SOL, TIB) (iv) serum Neuropeptide Y (v) serum substance P	(i) Compared with placebo-MA *p* < 0.05 (ii) Compared with placebo-MA *p* < 0.05 (iii–i) Compared with placebo-MA *p* < 0.05 (SOL, WASO, TST, SE) (iii–ii) Compared with placebo-MA *p* > 0.05 (TIB, ATs, REM-SOL) (iv) Compared with placebo-MA *p* < 0.05 (v) Compared with placebo-MA *p* < 0.05	No follow-up	No AEs
Chen et al. (40)▴	EA/*n* = 35 (11 M, 24 F) Paroxetine + Estazolam/*n* = 35 (9 M, 26 F)	NR	NR	DSM-V	NR	25 min/session, 5 sessions/week for 6 weeks (sparse wave, 2Hz)	Semi-standardized	EX-HN1, GB20, Gongxue (1.5 *Cun* below GB20), GV20, cluster needling on frontal region	Paroxetine 20 mg + Estazolam 1 mg/day for 6 weeks	(i) PSQI (ii) HAMD_17_ (iii) PHQ-15 (iv) total clinical effectiveness rate	(i) Compared with Paroxetine + Estazolam *p* < 0.01 (ii) Compared with Paroxetine + Estazolam *p* > 0.05 (iii) Compared with Paroxetine + Estazolam *p* < 0.01 (iv) Compared with Paroxetine + Estazolam *p* < 0.05	No follow-up	NR
Chen (41)▴	EA/*n* = 34 (14M, 20F) Sertraline/*n* = 33 (14M, 19F)	EA/36.52 ± 10.22 Sertraline/40.45 ± 11.39	EA/5.55 ± 1.59 m Sertraline/4.78 ± 1.59 m	ICD-10, DSM-V, CDTE-TCM	NR	30 min/session, 7 sessions/week for 4 weeks (continuous wave, 2 Hz, 0.6mA)	Fixed	EX-HN3, GV20, BL13, BL15, BL18, BL20, BL23	Sertraline 50 mg/day for 4 weeks	(i) PSQI (ii) HAMD_17_ (iii) total clinical effectiveness rate	(i) Compared with Sertraline *p* > 0.05 (ii) Compared with Sertraline *p* > 0.05 (iii) Compared with Sertraline *p* > 0.05	No follow-up	EA/*n* = 3 [hematoma] Sertraline/*n* = 15 [poor appetite (10), constipation (2), diarrhea (3)]
He (42)▴	MA/*n* = 32 (16 M, 16 F) Paroxetine/*n =* 32 (15 M, 17 F)	MA/45.38 ± 12.22 Paroxetine/42.25 ± 12.44	NR	ICD-10	NR	30 min/session, 7 sessions/week for 6 weeks	Fixed	GV20, HT7, PC6, Zhenjing, Shangen	Paroxetine 20 mg/day for 6 weeks	(i) PSQI (ii) SDS (iii) Total clinical effectiveness rate	(i) Compared with Paroxetine *p* > 0.05 (ii) Compared with Paroxetine *p* < 0.05 (iii) Compared with Paroxetine *p* > 0.05	(i) Lower PSQI in MA at 4-week follow-up (ii) Higher SDS in MA at 4-week follow-up (ii) Higher total clinical effectiveness rate in MA at 4-week follow-up	No AEs
Lin and Wang (43)▴	MA/*n* = 30 (14 M, 16 F) Escitalopram/*n* = 30 (16 M, 14 F)	MA/38.1 ± 10.3 Escitalopram/40.2 ± 9.1	MA/22.5 ± 12.9 m Escitalopram/20.5 ± 13.8 m	ICD-10	NR	30 min/session, 5 sessions/week for 4 weeks	Fixed	EX-HN1, EX-HN3, GV20, HT7, LR3, PC6, PC7	Escitalopram 10 mg/day for 4 weeks	(i) PSQI (ii) HAMD_17_ (iii) total clinical effectiveness rate	(i) Compared with Escitalopram *p* > 0.05 (ii) Compared with Escitalopram *p* > 0.05 (iii) Compared with Escitalopram *p* < 0.05	No follow-up	MA/*n* = 2 [fainting] Escitalopram/*n* = 9 [constipation (5), nausea (4)]
Lin (44)▴	EA/*n* = 24 (7 M, 17 F) Citalopram/*n* = 24 (5 M, 19 F)	EA/48.42 ± 13.42 Citalopram/47.58 ± 11.06	EA/9.38 ± 9.30 m Citalopram/15.88 ± 21.24	ICD-10	NR	20 min/session, 3 sessions/week for 6 weeks + 2 sessions/week for 6 weeks + 1 session/week for 12 weeks (intermittent wave, 40 Hz)	Fixed	EX-HN1, EX-HN3, GV20, HT7, PC6, SP6	Citalopram 30 mg/day for 24 weeks	(i) PSQI (ii) PSG (TST, ATs) (iii) SDS (iv) MADRS (v) SAS	(i) Compared with Citalopram *p* > 0.05 (ii) Compared with Citalopram *p* < 0.05 (TST, ATs) (iii) Compared with Citalopram *p* < 0.05 (iv) Compared with Citalopram *p* < 0.05 (v) Compared with Citalopram *p* < 0.01	No follow-up	No AEs
Liu (45)▴	MA/*n* = 30 (4 M, 26 F) Fluoxetine + Eszopiclone/*n* = 30 (3 M, 27 F)	MA/53.16 ± 8.32 Fluoxetine + Eszopiclone/54.43 ± 10.21	MA/2.87 ± 1.00 y Fluoxetine + Eszopiclone/3.06 ± 0.85 y	CCMD-3, CDTE-TCM	NR	30 min/session, 4 sessions/week for 7.5 weeks	Fixed	CV6, CV10, CV12, CV13, EX-HN3, GV20, GV24, PC6, ST25, ST36	(Fluoxetine 20 mg/day + Eszopiclone 2 mg/day) for 7.5 weeks	(i) PSQI (ii) HAMD_17_ (iii) Total clinical effectiveness rate (iv) Peripheral blood T-lymphocyte subsets (CD3, CD8, CD4/CD8, Ig M, Ig G)	(i) Compared with Fluoxetine + Eszopiclone *p* < 0.05 (ii) Compared with Fluoxetine + Eszopiclone *p* < 0.05 (iii) Compared with Fluoxetine + Eszopiclone *p* > 0.05 (iv) Compared with Fluoxetine + Eszopiclone *p* > 0.05	No follow-up	NR
Liu (46)▴	MA/*n* = 30 (16 M, 14 F) Clonazepam/*n* = 30 (18 M, 12 F)	MA/33.20 ± 9.85 Clonazepam/32.57 ± 10.17	MA/2.94 ± 5.60 m Clonazepam/2.88 ± 5.34 m	CCMD-3	Six TCM patterns (①②③④⑤⑥)	30 min/session, 6 sessions/week for 3 weeks	Fixed	0.5 *Cun* next to EX-HN1, 0.5 *Cun* up to EX-HN3, 0.5 *Cun* up to GB14, BL62, KI6, PC6, SP6	Clonazepam 1 mg/day, 2–4 days/week for 3 weeks	(i) PSQI (ii) SDS (iii) ChQoL (iv) total clinical effectiveness rate (v) SERS	(i) Compared with Clonazepam *p* < 0.01 (ii) Compared with Clonazepam *p* < 0.05 (iii) Compared with Clonazepam *p* < 0.01 (iv) Compared with Clonazepam *p* < 0.05 (v) Compared with Clonazepam *p* < 0.01	No significant difference in recurrence rate between two groups at 4- and 12-week follow-ups	MA/*n* = 2 [fainting] Clonazepam/*n* = 8 [dizziness (2), dry mouth (2), constipation (2), nausea and poor appetite (2)]
Wang and Liu (47)▴	MA/*n* = 45 (11 M, 34 F) Mirtazapine/*n* = 45 (13 M, 32 F)	MA/41.5 ± 4.6 Mirtazapine/39.1 ± 5.2	MA/4.9 ± 2.3 y Mirtazapine/5.1 ± 2.1 y	CCMD-3	NR	30 min/session, 3–4 sessions/week (once every other day) for 12 weeks	Fixed	EX-HN3, GV20, HT7, LR3, SP6, ST36	Mirtazapine 20 mg/day for 12 weeks	(i) PSQI (ii) HAMD_17_ (iii) Total clinical effectiveness rate (iv) AEs rate	(i) Compared with Mirtazapine *p* > 0.05 (ii) Compared with Mirtazapine *p* > 0.05 (iii) Compared with Mirtazapine *p* > 0.05 (iv) Compared with Mirtazapine *p* < 0.05	No follow-up	MA/*n* = 0 Mirtazapine/*n* = 13 [EDS (5), dry mouth (3), dizziness (3), fatigue (2)]
Wang et al. (48)▴	MA/*n* = 35 (17 M, 18 F) Mirtazapine/*n* = 37 (18 M, 19 F)	MA/45.8 ± 6.8 Mirtazapine/44.7 ± 5.1	MA/3.3 ± 0.8y Mirtazapine/3.3 ± 1.1y	DSM-IV, CDTE-TCM	②	30 min/session, 6 sessions/week for 4 weeks	Fixed	EX-HN3, GV20, HT7, LI4, LR3	Mirtazapine 30 mg/day for 4 weeks	(i) PSQI (ii) HAMD_24_ (iii) Serum 5-HT level (iv) Total clinical effectiveness rate (v) SERS	(i) Compared with Mirtazapine *p* < 0.05 (ii) Compared with Mirtazapine *p* > 0.05 (iii) Compared with Mirtazapine *p* > 0.05 (iv) Compared with Mirtazapine *p* > 0.05 (v) Compared with Mirtazapine *p* < 0.05	No follow-up	MA/*n* = 0 Mirtazapine/*n* = 5 [abnormal liver function (1), leukocytopenia (2), abnormal metabolism of blood fat (2)]
Ye and Yan (49)▴	MA/*n* = 40 (9 M, 31 F) Mirtazapine/*n* = 40 (12 M, 28 F)	NR	NR	CCMD-3	⑤⑦	30 min/session, 3-4 sessions/week (once every other day) for 12 weeks	Semi-standardized	EX-HN3, HT7, SP6	Mirtazapine 20 mg/day for 12 weeks	(i) PSQI (ii) HAMD_17_ (iii) Total clinical effectiveness rate	(i) Compared with Mirtazapine *p* > 0.05 (ii) Compared with Mirtazapine *p* > 0.05 (iii) Compared with Mirtazapine *p* > 0.05	No follow-up	MA/*n* = 0 Mirtazapine/*n* = 6 [EDS (1), dry mouth (1), weight gain (1), increased appetite (1), dizziness (1), fatigue (1)]
Liu and Li (50)▴	MA + Venlafaxine/*n* = 30 (9 M, 21 F) Venlafaxine/*n* = 31 (8 M, 23 F)	MA + Venlafaxine/43.2 ± 9.0 Venlafaxine/40.2 ±9.3	NR	ICD-10	②⑤⑦	5 sessions/week for 12 weeks	Semi-standardized	GV20, GV26, HT7, LR3, PC6, SP6	Venlafaxine 75 mg/day for 12 weeks	(i) PSQI (ii) HAMD (iii) HAMA	(i) Compared with Venlafaxine *p* < 0.05 (ii) Compared with Venlafaxine *p* < 0.05 (iii) Compared with Venlafaxine *p* < 0.05	No follow-up	NR
Liu et al. (51)▴	MA + Mirtazapine/*n* = 30 (14 M, 16 F) Mirtazapine/*n* = 30 (20 M, 10 F)	MA + Mirtazapine/41.33 ± 8.89 Mirtazapine/40.27 ± 9.72	MA + Mirtazapine/18.00 ± 9.49 m Mirtazapine/15.77 ± 7.93 m	CCMD-3	NR	30 min/session, 7 sessions/week for 4 weeks	Fixed	BL62, EX-HN1, GV20, HT7, KI6, LR3, PC6	Mirtazapine 30 mg/day for 4 weeks	(i) PSQI (ii) HAMD (iii) Total clinical effectiveness rate (iv) SERS	(i) Compared with Mirtazapine *p* < 0.05 (ii) Compared with Mirtazapine *p* < 0.05 (iii) Compared with Mirtazapine *p* < 0.05 (iv) Compared with Mirtazapine *p* < 0.05	No follow-up	Reflected by SERS scores
Sun et al. (52)▴	EA + Venlafaxine/*n* = 20 (13 M, 7 F) Venlafaxine/*n* = 20 (12 M, 8 F)	EA + Venlafaxine/32.5 ± 10.3 Venlafaxine/31.5 ± 11.4	EA + Venlafaxine/14.9 ± 3.6 m Venlafaxine/15.1 ± 6.35 m	CCMD-3	NR	30 min/session, 5 sessions/week for 2 weeks	Fixed	EX-HN3, GV20, PC6, ST36	Venlafaxine 150mg/d for 2 weeks	(i) PSG (TST, NREM%, REM%) (ii) HAMD (iii) Total clinical effectiveness rate	(i) TST, compared with Venlafaxine *p* < 0.05; NREM%, compared with Venlafaxine *p* < 0.05; REM%, compared with Venlafaxine *p* > 0.05 (ii) Compared with Venlafaxine *p* > 0.05 (iii) Compared with Venlafaxine *p* > 0.05	No follow-up	EA + Venlafaxine/*n* = 5 [fatigure and EDS (2), poor appetite (1), elevated blood pressure (2)] Venlafaxine/*n* = 6 [EDS (2), nausea (2), poor appetite (1), elevated blood pressure (1)]
Tan et al. (53)▴	MA + Paroxetine/*n* = 50 (23 M, 27 F) Paroxetine/*n* = 50 (21 M, 29 F)	MA + Paroxetine/40.42 ± 5.65 Paroxetine/40.63 ± 5.29	MA + Paroxetine/2.39 ± 0.65 y Paroxetine/2.51 ± 0.70 y	ICD-10	NR	30 min/session, 3–4 sessions/week (once every other day) for 6 weeks	Fixed	EX-HN1, HT7, LR3, SP6	Paroxetine 20 mg/day for 6 weeks	(i) PSQI (ii) HAMD_17_ (iii) Total clinical effectiveness rate (iv) SERS	(i) Compared with Paroxetine *p* < 0.05 (ii) Compared with Paroxetine *p* < 0.05 (iii) Compared with Paroxetine *p* < 0.05 (iv) Compared with Paroxetine *p* < 0.05	No follow-up	Reflected by SERS scores
Wang and Ai (54)▴	EA + Paroxetine/*n* = 45 (26 M, 19 F) Paroxetine/*n* = 35 (20 M, 15 F)	EA + Paroxetine/62.03 ± 4.11 Paroxetine/64.01 ± 4.41	EA + Paroxetine/2.59 ± 0.35 y Paroxetine/2.79 ± 0.26 y	CCMD-3	⑤⑧⑨	30 min/session, 7 sessions/week for 4 weeks (continuous wave)	Semi-standardized	EX-HN3, GV20, HT7, PC6, SP6, ST36	Paroxetine 20 mg/day for 4 weeks	(i) PSQI (ii) HAMD_17_ (iii) Total clinical effectiveness rate	(i) Compared with Paroxetine *p* < 0.05 (ii) Compared with Paroxetine *p* < 0.05 (iii) Compared with Paroxetine *p* < 0.01	No follow-up	NR
Min and Zhu (55)▴	MA + Paroxetine/*n* = 30 (12 M, 18 F) Paroxetine/*n* = 30 (14 M, 16 F)	MA + Paroxetine/36.1 ± 9.5 Paroxetine/35.4 ± 9.4	MA + Paroxetine/9.0 ± 1.2 m Paroxetine/8.8 ± 1.1 m	ICD-10	NR	3 sessions/week for 6 weeks	Semi-standardized	EX-HN3, GB20, GV14, GV16, GV20, PC6, SP6	Paroxetine 30 mg/day for 6 weeks	(i) PSQI (ii) HAMD_17_ (iii) SDS (iv) Total clinical effectiveness rate	(i) Compared with Paroxetine *p* < 0.05 (ii) Compared with Paroxetine *p* < 0.05 (iii) Compared with Paroxetine *p* < 0.05 (iv) Compared with Paroxetine *p* < 0.05	No follow-up	MA + Paroxetine/*n* = 6 [EDS (1), poor appetite and nausea (3), dry mouth (1), sweating (1)] Paroxetine/*n* = 7 [dizziness (1), poor appetite and nausea (4), sweating (2)]
Chung et al. (26)▾	EA/*n* = 60 (14 M, 46 F) Sham-EA/*n* = 60 (14 M, 46 F) Placebo-EA/*n* = 30 (3 M, 27 F)	EA/48.8 ± 9.9 Sham-EA/50.9 ± 9.5 Placebo-EA/47.4 ± 9.5	EA/8.7 ± 7.1 y Sham-EA/12.0 ± 11.4 y Placebo-EA/9.2 ± 8.4 y	DSM-IV	NR	30 min/session, 3 sessions/week for 3 weeks (square wave, 4-Hz)	Fixed	EX, EX-HN1, EX-HN3, GV20, HT7, PC6, SP6, TF_4_	Sham-EA (superficial acupuncture) or placebo-EA (Streitberger acupuncture) on non-acupoins, 30 min/session, 3 sessions/week for 3 weeks	(i) PSQI (ii) ISI (iii) HAMD_17_ (iv) SDS (v) Actigraphy (SOL, TST, WASO, SE) (vi) Sleep diary (SOL, TST, WASO, SE, sleep quality) (vii) ESS (viii) HAMA (ix) MFI (x) CTRS	(i–i) Compared with sham-EA *p* > 0.05 (i–ii) Compared with placebo-EA *p* > 0.05 (ii–i) compared with sham-EA *p* > 0.05 (ii–ii) Compared with placebo-EA *p* > 0.05 (iii–i) Compared with sham-EA *p* > 0.05 (iii–ii) Compared with placebo-EA *p* > 0.05 (iv–i) Compared with sham-EA *p* > 0.05 (iv–ii) Compared with placebo-EA *p* > 0.05 (v–i) Compared with sham-EA *p* > 0.05 (v–ii) Compared with placebo-EA *p* > 0.05 (vi–i) Compared with sham-EA *p* > 0.05 (vi–ii) Compared with placebo-EA *p* > 0.05 (vii–i) Compared with sham-EA *p* > 0.05 (vii–ii) Compared with placebo-EA *p* > 0.05 (viii–i) Compared with sham-EA *p* > 0.05 (viii–ii) Compared with placebo-EA *p* > 0.05 (ix–i) Compared with sham-EA *p* > 0.05 (ix–ii) Compared with placebo-EA *p* > 0.05 (x–i) Compared with sham-EA *p* > 0.05 (x–ii) Compared with placebo-EA *p* > 0.05	The results at 5-week follow-up were largely consistent with the results at post-treatment	EA/*n* = 41 [pain at acupoints (19), headache (8), fatigue (8), dizziness (3), nausea (3)] Sham-EA/*n* = 18 [pain at acupoints (6), headache (7), fatigue (4), nausea (1)] Placebo-EA/*n* = 6 [pain at acupoints (1), headache (3), dizziness (1), nausea (1)]
Yeung et al. (37)▾	EA/*n* = 26 (6 M, 20 F) Sham-EA/*n* = 26 (7 M, 19 F) Placebo-EA/*n* = 26 (3 M, 23 F)	EA/47.5 ± 8.5 Sham-EA/46.7 ± 9.7 Placebo-EA/50.1 ± 9.1	EA/8.9 ± 10.1 y Sham-EA/11.5 ± 9.4 y Placebo-EA/12.6 ± 8.6 y	DSM-IV	NR	30 min/session, 3 sessions/week for 3 weeks (square wave, 4 Hz)	Fixed	EX, EX-HN1, EX-HN3, GV20, TF_4_	Sham-EA (superficial acupuncture) on non-acupoints or placebo-EA (Streitberger acupuncture) on the same acupoints as those in the EA group, 30 min/session, 3 sessions/week for 3 weeks	(i) PSQI (ii) ISI (iii) HAMD_17_ (iv) SDS (v) Actigraphy (SOL, TST, WASO, SE) (vi) sleep diary (SOL, TST, WASO, SE, sleep quality) (vii) CTRS	(i–i) Compared with sham-EA *p* > 0.05 (i–ii) Compared with placebo-EA *p* < 0.05 (ii–i) Compared with sham-EA *p* > 0.05 (ii–ii) Compared with placebo-EA *p* < 0.05 (iii–i) Compared with sham-EA *p* > 0.05 (iii–ii) Compared with placebo-EA *p* > 0.05 (iv–i) Compared with sham-EA *p* > 0.05 (iv–ii) Compared with placebo-EA *p* > 0.05 (v–i) Compared with sham-EA *p* > 0.05; (v–ii) compared with placebo-EA *p* > 0.05; (vi–i) Compared with sham-EA *p* > 0.05; (vi–ii) SE, compared with placebo-EA *p* < 0.05; other parameters compared with placebo-EA *p* > 0.05; (vii–i) Compared with sham-EA *p* > 0.05; (vii–ii) Compared with placebo-EA *p* > 0.05	The results at 4-week follow-up were largely consistent with the results at post-treatment	EA/*n* = 3 [headache (2), dizziness (1)] Sham-EA/*n* = 6 [worsening of insomnia (1), hand numbness (2), hematoma (1), palpitation (1), pain at acupoints (1)] Placebo-EA/*n* = 5 [headache (2), dizziness (2), hand numbness (1)]

*TCM patterns [① hyperactivity of the fire of the Heart, ② fire derived from stagnation of Liver-Qi, ③ interior disturbance of phlegm-heat, ④ hyperactivity of fire due to Yin deficiency, ⑤ deficiency of both Heart and Spleen, ⑥ deficiency of Heart-Qi and Gallbladder-Qi, ⑦ depression of Liver-Qi; ⑧ Liver depression and Spleen deficiency; ⑨ Spleen-Kidney Yang deficiency]; type of insomnia [insomnia as a major symptom of active depression (▴); insomnia as a residual symptom of previous or partial-remission depression (▾)]. For semi-standardized acupuncture prescriptions, only major/core acupoints are listed*.

*NR, no report; MA, manual acupuncture; EA, electroacupuncture; ICD-10, International Classification of Diseases (10th edition); CCMD-3, Chinese Classification of Mental Disorders (third edition); DSM-IV, Diagnostic and Statistical Manual of Mental Disorders (fourth edition); DSM-V, Diagnostic and Statistical Manual of Mental Disorders (fifth edition); CDTE-TCM, Criteria of Diagnosis and Therapeutic Effect of Diseases and Syndromes in TCM; PSQI, Pittsburgh Sleep Quality Index; ISI, Insomnia Severity Index; ESS, Epworth Sleepiness Scale; HAMD, Hamilton Depression Scale; SDS, Self-rating Depression Scale; SAS, Self-rating Anxiety Scale; MADRS, Montgomery-Asberg Depression Rating Scale; HAMA, Hamilton Anxiety Scale; PHQ-15, Patient Health Questionnaire-15; MFI, Multidimensional Fatigue Inventory; ChQoL, Chinese quality-of-life instrument; SERS, Asberg Side Effects Rating Scale; CTRS, Credibility of Treatment Rating Scale; PSG, polysomnography; TIB, time in bed; SOL, sleep-onset latency; WASO, wake after sleep onset; ATs, awakening times; TST, total sleep time; SE, sleep efficiency; REM, rapid eye movement sleep; NREM, non-rapid eye movement sleep; REM-SOL, REM sleep-onset latency; 5-HT, 5-hydroxytryptamine; AEs, adverse events; EDS, excessive daytime sleepiness; BL13, Feishu; BL15, Xinshu; BL18, Ganshu; BL20, Pishu; BL23, Shenshu; BL62, Shenmai; CV6, Qihai, CV10, Xiawan; CV12, Zhongwan; CV13, Shangwan; EX, Anmian; EX-HN1, Sishencong; EX-HN3, Yintang; GB13, Benshen; GB14, Yangbai; GB20, Fengchi; GV11, Shendao; GV14, Dazhui; GV16, Fengfu; GV20, Baihui; GV24, Shenting; GV26, Shuigou; HT7, Shenmen; KI6, Zhaohai; LI4, Hegu; LR3, Taichong; PC6, Neiguan; PC7, Daling; SP6, Sanyingjiao; ST25, Tianshu; ST36, Zusanli; TF_4_, Ear Shenmen*.

Acupuncture treatment was delivered daily to approximately twice (1.75 times) per week for 3 to 24 weeks. The duration of each treatment session (needle retention time) ranged from 20 to 30 min. Sixteen RCT trials adopted a standardized treatment protocol, with a fixed selection of acupoints administered for each patient assigned to the same group at each acupuncture session. The remaining five trials (40, 49, 50, 54, 55) adopted a semi-standardized treatment protocol consisting of semi-fixed acupoints, including a predefined set of core/major acupoints utilized in combination with acupoints selected on the basis of each patient's symptoms or TCM pattern to which he/she presents. Individualized treatment protocol was not adopted in any RCT. The selection of acupoints varied and included acupoints located on the head, extremities, and abdomen. Among them, the five most frequently utilized acupoints were Baihui (GV20), Yintang (EX-HN3), Shenmen (HT7), Neiguan (PC6), and Sanyinjiao (SP6). In the five trials that included sham-/placebo-acupuncture (26, 27, 37–39), the sham-acupuncture was performed by superficially piercing a region close to but not an acupoint with a needle, and the placebo-acupuncture was performed by placing a Streitberger needle on the skin surface (not penetrating the skin) at the same acupoint as that of real acupuncture or at a region beside an acupoint (Table 1).

The major outcome measures and the time points at which they were evaluated are summarized in Table 2. All except one RCT (52) adopted PSQI global scores as the primary outcome measurement tool in assessing sleep quality and quantity. Six RCTs recorded changes in objective sleep parameters [e.g., total sleep time (TST), SE, SOL, wake after sleep onset (WASO), awakening times (ATs), and percentage of rapid eye movement sleep (REM) and non-REM] among patients at pre- and post-treatment, by either PSG (39, 44, 52) or actigraphy (26, 27, 37). In the assessment of depressed mood, 14 RCTs used the HAMD only (38–41, 43, 45, 47–54); three RCTs used the SDS only (42, 44, 46); and the remaining four RCTs included both scales (26, 27, 37, 55). HAMD-17 was used in 13 RCTs (26, 27, 37, 39–41, 43, 45, 47, 49, 53–55) and HAMD-24 in two (38, 48). The remaining three trials (50–52) did not depict which version of HAMD was utilized. Five studies (26, 27, 37, 42, 46) reported follow-up data from 1 to 12 weeks after the end of treatment (Table 1).

**Table 2:** Trends of major outcomes for insomnia and depression in acupuncture (OR acupuncture + antidepressant and/or hypnotic) and comparison with controls in each study.

**References**	**Type of insomnia**	**Comparison**		**Outcome measures for insomnia**						**Outcome measures for depression**
				**Subjective outcome**	**Objective outcome (data from PSG or actigraphy)**					**HAMD or SDS**
				**PSQI or ISI**	**TST**	**SOL**	**SE**	**WASO**	**ATs**	
Yin et al. (27)	Major	*Vs*. the same group at different time points	4-week treatment *vs*. pre-treatment	↓	↑	Ø	↑	Ø	↓	↓
			Post-treatment *vs*. pre-treatment	↓	↑	Ø	↑	Ø	↓	↓
			4-week follow-up *vs*. pre-treatment	↓	↑	Ø	↑	Ø	↓	↓
		Acup *vs*. sham-/placebo-Acup at the same time point	4-week treatment	<	*Vs*. sham (–), *Vs*. placebo, >	Ø	*Vs*. sham (–), *Vs*. placebo, >	Ø	(–)	<
			Post-treatment	<	>	Ø	>	Ø	(–)	<
			4-week follow-up	<	*Vs*. sham (–), *Vs*. placebo, >	Ø	*Vs*. sham (–), *Vs*. placebo, >	Ø	(–)	<
Qin et al. (38)	Major	*Vs*. the same group at different time points	Post-treatment *vs*. pre-treatment	↓	Ø	Ø	Ø	Ø	Ø	↓
		Acup *vs*. placebo-Acup at the same time point	Post-treatment	<	Ø	Ø	Ø	Ø	Ø	<
Zhao et al. (39)	Major	*Vs*. the same group at different time points	Post-treatment *vs*. pre-treatment	↓	↑	↓	↑	↓	(–)	↓
		Acup *vs*. placebo-Acup at the same time point	Post-treatment	<	>	<	>	<	(–)	<
Chen et al. (40)	Major	*Vs*. the same group at different time points	Post-treatment *vs*. pre-treatment	↓	Ø	Ø	Ø	Ø	Ø	↓
		Acup *vs*. antidepressant + hypnotic at the same time point	Post-treatment	<	Ø	Ø	Ø	Ø	Ø	(–)
Chen (41)	Major	*Vs*. the same group at different time points	2-week treatment *vs*. pre-treatment	↓	Ø	Ø	Ø	Ø	Ø	↓
			Post-treatment *vs*. pre-treatment	↓	Ø	Ø	Ø	Ø	Ø	↓
			Post-treatment *vs*. 2-week treatment	↓	Ø	Ø	Ø	Ø	Ø	↓
		Acup *vs*. antidepressant at the same time point	2-week treatment	>	Ø	Ø	Ø	Ø	Ø	>
			Post-treatment	(–)	Ø	Ø	Ø	Ø	Ø	(–)
He (42)	Major	*Vs*. the same group at different time points	Post-treatment *vs*. pre-treatment	↓	Ø	Ø	Ø	Ø	Ø	↓
			4-week follow-up *vs*. post-treatment	(–)	Ø	Ø	Ø	Ø	Ø	(–)
		Acup *vs*. antidepressant at the same time point	Post-treatment	(–)	Ø	Ø	Ø	Ø	Ø	>
			4-week follow-up	<	Ø	Ø	Ø	Ø	Ø	<
Lin and Wang (43)	Major	*Vs*. the same group at different time points	Post-treatment *vs*. pre-treatment	↓	Ø	Ø	Ø	Ø	Ø	↓
		Acup vs. antidepressant at the same time point	Post-treatment	(–)	Ø	Ø	Ø	Ø	Ø	(–)
Lin (44)	Major	*Vs*. the same group at different time points	12-week treatment *vs*. pre-treatment	↓	↑	(–)	(–)	(–)	(–)	↓
			Post-treatment *vs*. pre-treatment	↓	↑	(–)	(–)	(–)	↓	↓
		Acup *vs*. antidepressant at the same time point	12-week treatment	(–)	(–)	Ø	Ø	Ø	<	>
			Post-treatment	(–)	(–)	Ø	Ø	Ø	<	>
Liu (45)	Major	*Vs*. the same group at different time points	Post-treatment *vs*. pre-treatment	↓	Ø	Ø	Ø	Ø	Ø	↓
		Acup *vs*. antidepressant + hypnotic at the same time point	Post-treatment	<	Ø	Ø	Ø	Ø	Ø	<
Liu (46)	Major	*Vs*. the same group at different time points	Post-treatment *vs*. pre-treatment	↓	Ø	Ø	Ø	Ø	Ø	↓
		Acup *vs*. hypnotic at the same time point	Post-treatment	<	Ø	Ø	Ø	Ø	Ø	<
Wang and Liu (47)	Major	*Vs*. the same group at different time points	Post-treatment *vs*. pre-treatment	↓	Ø	Ø	Ø	Ø	Ø	↓
		Acup *vs*. antidepressant at the same time point	Post-treatment	(–)	Ø	Ø	Ø	Ø	Ø	(–)
Wang et al. (48)	Major	*Vs*. the same group at different time points	Post-treatment *vs*. pre-treatment	↓	Ø	Ø	Ø	Ø	Ø	↓
		Acup *vs*. antidepressant at the same time point	Post-treatment	<	Ø	Ø	Ø	Ø	Ø	(–)
Ye and Yan (49)	Major	*Vs*. the same group at different time points	4-week treatment *vs*. pre-treatment	↓	Ø	Ø	Ø	Ø	Ø	↓
			8-week treatment *vs*. pre-treatment	↓	Ø	Ø	Ø	Ø	Ø	↓
			Post-treatment *vs*. pre-treatment	↓	Ø	Ø	Ø	Ø	Ø	↓
		Acup *vs*. antidepressant at the same time point	4-week treatment	(–)	Ø	Ø	Ø	Ø	Ø	(–)
			8-week treatment	(–)	Ø	Ø	Ø	Ø	Ø	(–)
			Post-treatment	(–)	Ø	Ø	Ø	Ø	Ø	(–)
Liu and Li (50)	Major	*Vs*. the same group at different time points	Post-treatment *vs*. pre-treatment	↓	Ø	Ø	Ø	Ø	Ø	↓
		Acup + antidepressant *vs*. antidepressant at same time point	Post-treatment	<	Ø	Ø	Ø	Ø	Ø	<
Liu et al. (51)	Major	*Vs*. the same group at different time points	Post-treatment *vs*. pre-treatment	↓	Ø	Ø	Ø	Ø	Ø	↓
		Acup + antidepressant *vs*. antidepressant at the same time point	Post-treatment	<	Ø	Ø	Ø	Ø	Ø	<
Sun et al. (52)	Major	*Vs*. the same group at different time points	1-week treatment *vs*. pre-treatment	Ø	Ø	Ø	Ø	Ø	Ø	↓
			Post-treatment *vs*. pre-treatment	Ø	↑	Ø	Ø	Ø	Ø	↓
		Acup + antidepressant *vs*. antidepressant at the same time point	1-week treatment	Ø	Ø	Ø	Ø	Ø	Ø	>
			post-treatment	Ø	>	Ø	Ø	Ø	Ø	(–)
Tan et al. (53)	Major	*Vs*. the same group at different time points	Post-treatment *vs*. pre-treatment	↓	Ø	Ø	Ø	Ø	Ø	↓
		Acup + antidepressant *vs*. antidepressant at the same time point	Post-treatment	<	Ø	Ø	Ø	Ø	Ø	<
Wang and Ai (54)	Major	*Vs*. the same group at different time points	1-week treatment *vs*. pre-treatment	↓	Ø	Ø	Ø	Ø	Ø	↓
			2-week treatment *vs*. pre-treatment	↓	Ø	Ø	Ø	Ø	Ø	↓
			3-week treatment *vs*. pre-treatment	↓	Ø	Ø	Ø	Ø	Ø	↓
			Post-treatment *vs*. pre-treatment	↓	Ø	Ø	Ø	Ø	Ø	↓
		Acup + antidepressant *vs*. antidepressant at same time-point	1-week treatment	<	Ø	Ø	Ø	Ø	Ø	<
			2-week treatment	<	Ø	Ø	Ø	Ø	Ø	<
			3-week treatment	<	Ø	Ø	Ø	Ø	Ø	<
			Post-treatment	<	Ø	Ø	Ø	Ø	Ø	<
Min and Zhu (55)	Major	*Vs*. the same group at different time points	Post-treatment *vs*. pre-treatment	↓	Ø	Ø	Ø	Ø	Ø	↓
		Acup + antidepressant *vs*. antidepressant at the same time point	Post-treatment	<	Ø	Ø	Ø	Ø	Ø	<
Chung et al. (26)	Residual	*Vs*. the same group at different time points	1-week post-treatment *vs*. pre-treatment	(–)	(–)	(–)	(–)	(–)	Ø	(–)
			5-week follow-up *vs*. pre-treatment	(–)	(–)	(–)	(–)	(–)	Ø	(–)
		Acup *vs*. sham-/placebo-Acup at the same time point	1-week post-treatment	(–)	(–)	(–)	(–)	(–)	Ø	(–)
			5-week follow-up	(–)	(–)	(–)	(–)	(–)	Ø	(–)
Yeung et al. (37)	Residual	*Vs*. the same group at different time points	1-week post-treatment *vs*. pre-treatment	↓	(–)	(–)	(–)	(–)	Ø	(–)
			4-week follow-up *vs*. pre-treatment	↓	(–)	(–)	(–)	(–)	Ø	(–)
		Acup *vs*. sham-/placebo-Acup at the same time point	1-week post-treatment	*Vs*. sham (–), *vs*. placebo, <	(–)	(–)	(–)	(–)	Ø	(–)
			4-week follow-up	*Vs*. sham (–), *vs*. placebo, <	(–)	(–)	(–)	(–)	Ø	(–)

*↑, statistically increase; ↓, statistically decrease; >, statistically higher/longer/more; <, statistically lower/shorter/less; (–), no statistical changes/no statistical difference; Ø, N/A or no data; Type of insomnia (major, insomnia as a major symptom of active depression; residual, insomnia as a residual symptom of previous or partial-remission depression)*.

*Acup, acupuncture; PSG, polysomnography; PSQI, Pittsburgh Sleep Quality Index; ISI, Insomnia Severity Index; HAMD, Hamilton Depression Scale; SDS, Self-rating Depression Scale; SOL, sleep-onset latency; WASO, wake after sleep onset; ATs, awakening times; TST, total sleep time; SE, sleep efficiency*.

Sixteen studies (26, 27, 37, 39, 41–44, 46–49, 51–53, 55) reported detailed information of AEs. The most frequently reported AEs among participants receiving both acupuncture treatment and placebo-/sham-acupuncture treatment were hand numbness and/or pain at acupoints [(22/116) in acupuncture; (11/142) in placebo-/sham-acupuncture], fatigue [(8/60) in acupuncture; (4/90) in placebo-/sham-acupuncture], and headache [(10/86) in acupuncture; (12/142) in placebo-/sham-acupuncture]. The most relevant AEs associated with antidepressant and/or hypnotics were gastrointestinal symptoms, such as poor appetite, diarrhea, and constipation (38/183); abnormal blood or biochemical indicators such as abnormal liver function, leukocytopenia, or abnormal metabolism of blood fat (5/37); and excessive daytime sleepiness (8/105) (Appendix 3).

### Study Quality Evaluation

**Figure 2:**
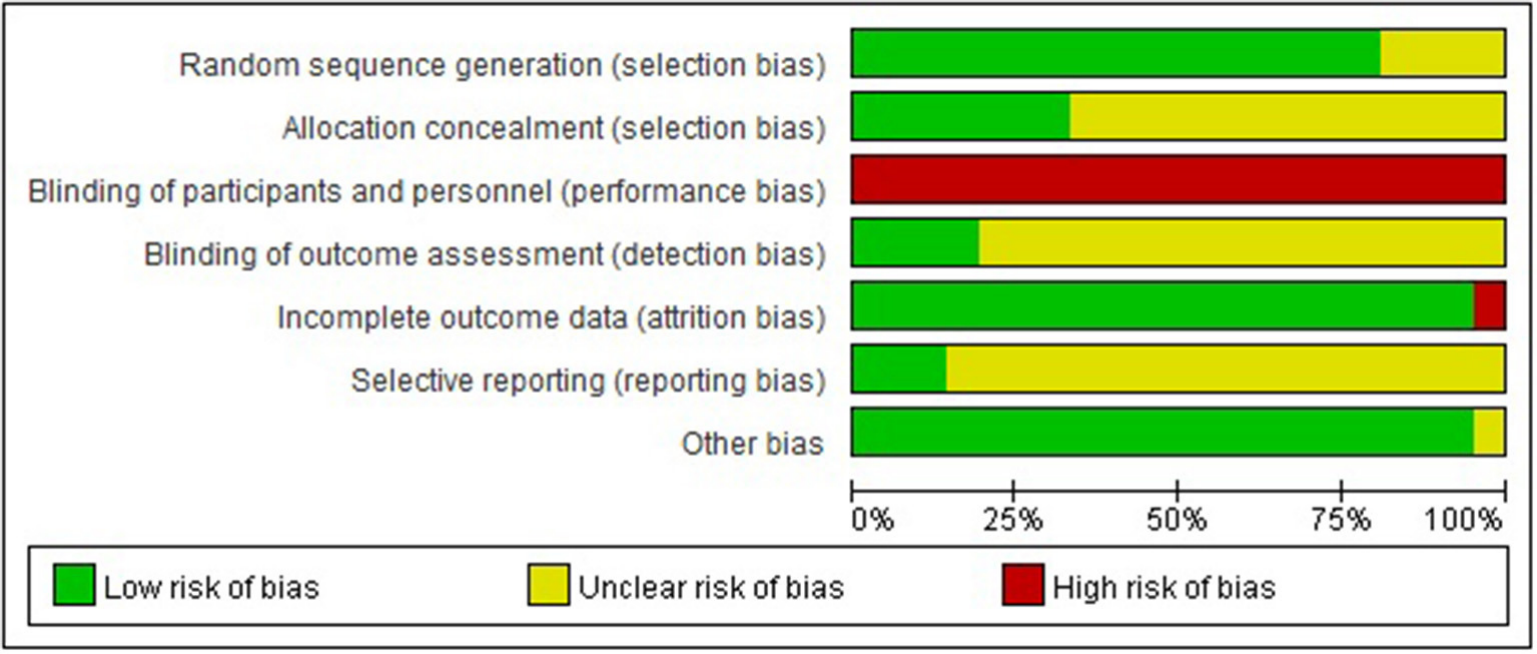
Risk-of-bias graph. Other biases are assessed based on baseline balance.

Seventeen out of 21 trials provided an adequate description of the process and method of randomization (26, 27, 37–40, 42–46, 48, 51–55), while four trials (41, 47, 49, 50) only mentioned that an RCT design was fulfilled in the trial but did not adequately describe the randomization approach and procedure. All except for seven trials (26, 27, 37, 39, 40, 44, 46) were judged as unclear risk of bias for allocation concealment. Valid allocation concealment was achieved in those seven studies by the method of “opaque envelopes.” Less than one-fifth of RCTs (26, 27, 37, 48) reported that the outcome evaluator was blinded. Incomplete outcome data were judged as low risk of bias in 20 studies. Among them, 12 studies (38–40, 46–52, 54, 55) reported no withdrawal of participants; three studies (26, 27, 37) addressed the dropout case and missing data with sound statistical methods such as intention-to-treat analysis; five studies (41–43, 45, 53) directly excluded data from those dropout cases, while reporting that the number of dropout cases was <10% of the initial samples, which is within the controllable range. Only one study (44) was evaluated as being a high risk of bias in this domain because the number of dropout cases reached 20% and no statistical treatment was assigned to those cases. For the item of selective outcome reporting, three trials (26, 27, 37) were appraised as being a low risk of bias due to accessible pre-registration information of the trial. The remaining studies were rated as being an unclear risk of bias because of unavailable protocols or because there was insufficient evidence and information to permit a clear judgment. “Blinding of personnel (acupuncturist)” in all studies was rated as a high risk of bias due to the nature of acupuncture. Acupuncture techniques require manipulation by a qualified professional to implement; blinding for acupuncturists hence is not feasible. It is reassuring that either placebo-acupuncture (Streitberger needles) or sham-acupuncture (superficial acupuncture at non-acupoints) was introduced as reasonable placebo in all five RCTs (26, 27, 37–39), where non-pharmaceuticals were used as control, to blind patients and improve the results reliability. “Blinding of participants (patients)” might be challenging in the remaining RCTs as they addressed the comparison between acupuncture/acupuncture + pharmacotherapy and pharmacotherapy alone. All studies addressed baseline balance adequately (Figure 2, Appendices 4, 5). In compliance with the modified Jadad system, six studies (26, 27, 37–39, 48) had high methodological quality as indicated by a score over 3 points; five studies (41, 44, 47, 49, 50) had low methodological quality as indicated by a score below 3 points; and the remaining studies with a score of 3 points were judged as being of moderate methodological quality. The average modified Jadad score of all 21 studies was 3.2 (Appendix 4).

Appendix 6 summarizes the details about acupuncture per STRICTA guidelines. Traditional Chinese acupuncture was used in all 21 trials, and treatment was provided in accordance with the TCM theory. As the core part of acupuncture therapy, the needling details were not clearly described in some RCTs. For instance, the exact depth of insertion was presented in detail in only nine trials (26, 27, 39, 41, 43–46, 48), and five studies (43, 45, 50, 52, 55) did not manifest the needle type used. All except two RCTs (50, 55) reported needle retention time which ranged from 20 to 30 min. Setting of treatment was described in only three trials (26, 27, 37). Similarly, only these three trials clearly presented the acupuncturist's background.

### Analysis of Outcome Measures

The qualitative and quantitative analyses for outcome measures in the 21 included studies were classified into two categories according to the types of DI. In the first category (the therapeutic effect of acupuncture on insomnia as a major symptom of active depression), we further split the analyses into three sections: (1) acupuncture *vs*. placebo-/sham-acupuncture (*n* = 3 RCTs); (2) acupuncture *vs*. Western medication (antidepressant and/or hypnotic) (*n* = 10 RCTs); and (3) acupuncture combined with Western medication *vs*. Western medication (*n* = 6 RCTs). In the second category (the therapeutic effect of acupuncture on insomnia as a residual symptom of previous or partially remitted depression), we only qualitatively described the results because there were not enough RCTs (*n* < 3) to conduct meta-analysis (Appendix 7).

#### Effect of Acupuncture on Insomnia as a Major Symptom of Active Depression

##### Acupuncture *vs*. Placebo-/Sham-Acupuncture

**Figure 3:**
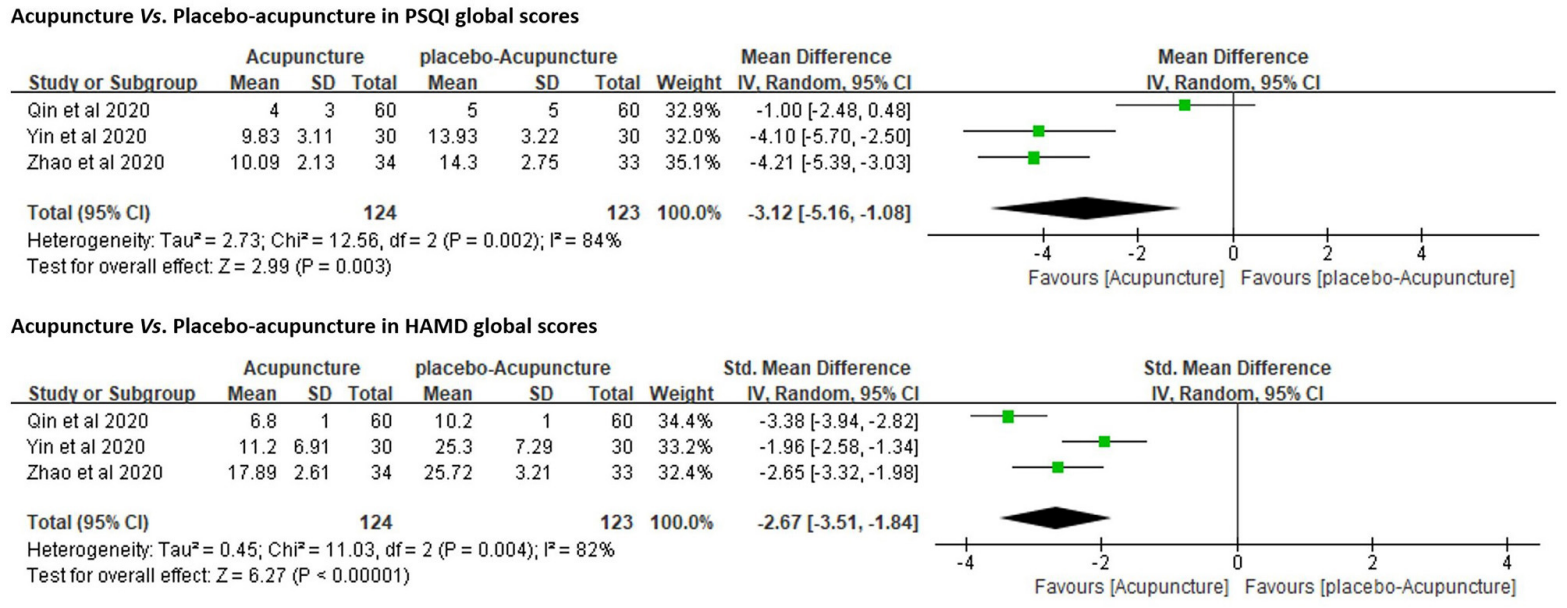
Forest plots of acupuncture *vs*. placebo-acupuncture in PSQI and HAMD.

Three RCTs (27, 38, 39) (*n* = 277) were under this category and addressed the comparison between verum- and placebo-acupuncture (by using Streitberger needles). They all included PSQI as the primary outcome measure. Due to the high heterogeneity (*p* < 0.01, *I*^2^ = 84%), a random-effects model was used. The results favored acupuncture in reducing PSQI global scores [MD = −3.12, 95%CI (−5.16, −1.08), *p* < 0.01]. These three RCTs also adopted HAMD (17- or 24-item version) to explore the regulating effect of verum- or placebo-acupuncture on depressed mood. The results favored acupuncture in reducing HAMD global scores as well [SMD = −2.67, 95%CI (−3.51, −1.84), *p* < 0.01; Figure 3]. One of the three RCTs also included sham-acupuncture (superficial acupuncture at non-acupoints) as another parallel control and came to the same conclusion (27).

In addition to subjective sleep quality and quantity, objective sleep parameters were shown to be improved with acupuncture but not placebo-/sham-acupuncture in two of the three trials (27, 39). In accordance with the records of actigraphy or PSG, acupuncture significantly shortened sleep-onset latency (SOL), prolonged the total sleep time (TST), elevated sleep efficiency (SE), and reduced wake after sleep onset (WASO) in comparison with placebo-/sham-acupuncture. Neither acupuncture nor placebo-/sham-acupuncture reduced the number of ATs (Table 2).

Only one RCT included follow-up (27). At the 4-week follow-up, the advantage of acupuncture over placebo-/sham-acupuncture in reducing PSQI and HAMD scores as well as in increasing TST and SE remained significant (Table 2).

##### Acupuncture *vs*. Western Medication

Ten RCTs (*n* = 668) were included in this comparison. Meta-analyses were carried out for four indices, namely, PSQI (global scores and six dimensions of PSQI), HAMD, SDS, and total clinical effectiveness rate. Subgroup analysis, sensitivity analysis, and meta-regression analysis were adopted to investigate the sources of heterogeneity where necessary. We did not perform meta-analysis for other outcome measures because there were fewer than three studies for each of them (Appendix 7).

##### Data Synthesis

###### PSQI Global Scores.

**Figure 4:**
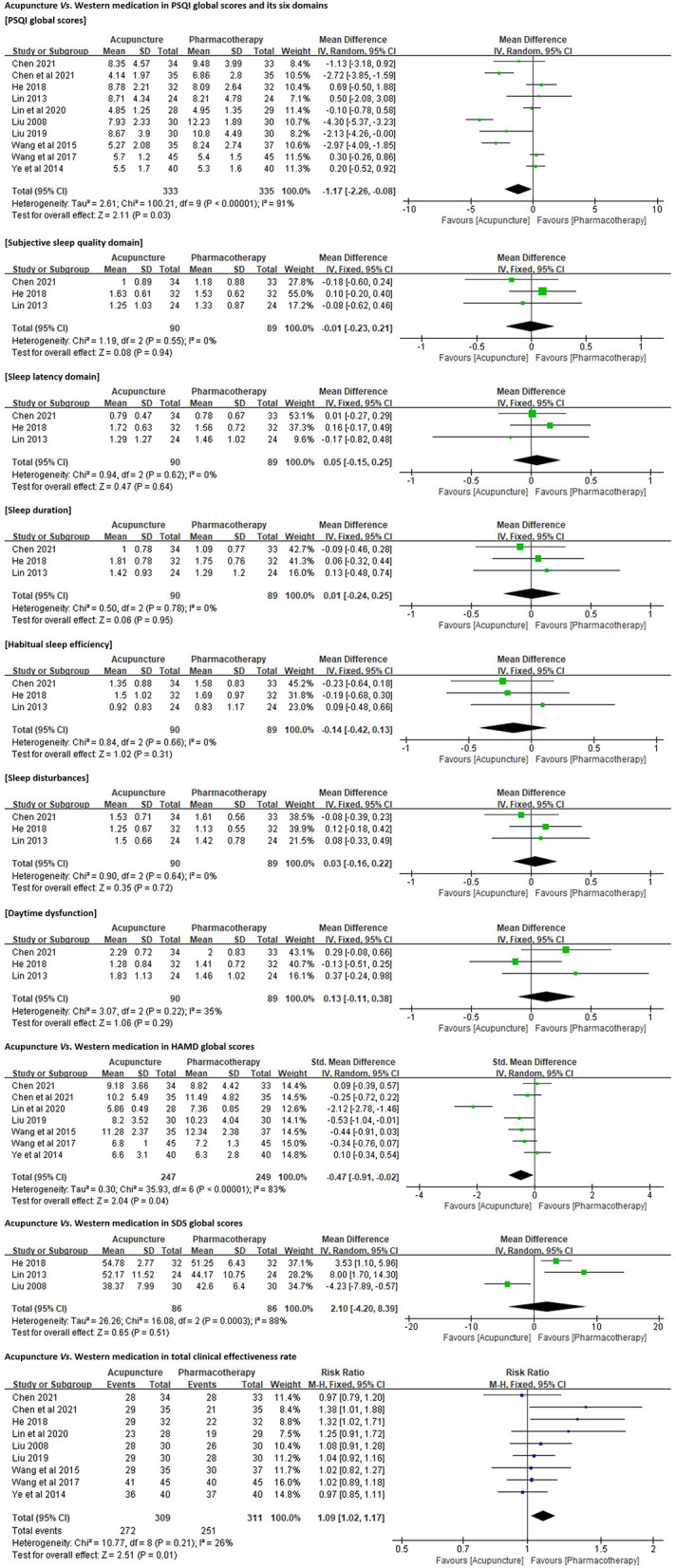
Forest plots of acupuncture *vs*. Western medication in PSQI, HAMD, SDS, and total clinical effectiveness rate.

All 10 trials employed PSQI as the primary outcome measure. Due to the high heterogeneity (*p* < 0.01, I^2^ = 91%), a random-effects model was used. The results favored acupuncture in reducing PSQI global scores [MD = −1.17, 95% CI (−2.26, −0.08), *p* = 0.03; Figure 4].

###### Six Dimensions of PSQI.

Three of the 10 trials (n = 179) (41, 42, 44) reported the scores for each domain of PSQI in detail. No significant differences were identified between acupuncture and antidepressant (paroxetine, sertraline, or citalopram) in improving subjective sleep quality [MD = −0.01, 95% CI (−0.23, 0.21), *p* = 0.94], shortening sleep latency [MD = 0.05, 95% CI (−0.15, 0.25), *p* = 0.64], increasing sleep duration [MD = 0.01, 95% CI (−0.24, 0.25), *p* = 0.95] and habitual sleep efficiency [MD = −0.14, 95% CI (−0.42, 0.13), *p* = 0.31], and ameliorating sleep disturbances [MD = 0.03, 95% CI (−0.16, 0.22), *p* = 0.72] and daytime dysfunction [MD = 0.13, 95% CI (−0.11, 0.38), *p* = 0.29; Figure 4].

###### HAMD Global Scores.

Seven trials (n = 496) (40, 41, 43, 45, 47–49) employed either 17-item or 24-item HAMD to quantify the participants' depression symptoms. A random-effects model was used because of the high heterogeneity (*p* < 0.01, I^2^ = 83%). The results favored acupuncture in reducing HAMD global scores [SMD = −0.47, 95% CI (−0.91, −0.02), *p* = 0.04; Figure 4].

###### SDS Global Scores.

Three trials (n = 172) (42, 44, 46) employed SDS to assess the participants' depression levels. The resulting assessment was inconsistent with our analysis for HAMD. No significant differences were identified between acupuncture and antidepressant or hypnotic in reducing SDS global scores [MD = 2.10, 95% CI (−4.20, 8.39), *p* = 0.51; Figure 4].

###### Total Clinical Effectiveness Rate.

All (n = 620) except one trial (44) compared the clinical effectiveness rates between acupuncture and pharmacotherapy for DI (Appendix 8). Pooled analysis results favored acupuncture in increasing the total effectiveness rate for DI [RR = 1.09, 95% CI (1.02, 1.17), *p* = 0.01; Figure 4].

##### Subgroup Analysis

On the basis of different acupuncture methods (MA or EA), principles of acupuncture prescription (fixed or semi-standardized), acupuncture treatment frequency [high (≥5 sessions per week) or low (<5 sessions per week)], and needle retention time (≥30 or <30 min), different medication in control groups (antidepressant alone, hypnotic alone, or antidepressant + hypnotic), and different versions of HAMD (17-item or 24-item), we conducted subgroup analyses under PSQI and HAMD. The significant interaction effect was identified between different acupuncture treatment frequencies under PSQI (χ^2^ statistic 4.15, df = 1, *p* = 0.04). Acupuncture with high-frequency treatment sessions more significantly reduced PSQI scores in comparison with pharmacotherapy [MD = −1.76, 95% CI (−3.40, −0.13), *p* = 0.03]. In contrast, there was no significant difference between acupuncture with low-frequency treatment sessions and pharmacotherapy in reducing PSQI scores [MD = 0.07, 95% CI (−0.59, 0.73), *p* = 0.84]. Notably, no significant interaction effect was found between different acupuncture treatment frequencies under HAMD (χ^2^ statistic 0.82, df = 1, *p* = 0.36). We also identified the significant interaction effect between different drugs in controls under PSQI (χ^2^ statistic 33.66, df = 2, *p* < 0.01), while only one trial was included in the hypnotic subgroup and only two trials were included in the antidepressant + hypnotic subgroup. No interaction was identified in any other subgroups, which means the heterogeneity still could not be fully explained (Appendix 9).

There was also an interesting discovery about PSQI. When all 10 trials were pooled for effect size, acupuncture showed better effects than psychotropic drugs (antidepressant and/or hypnotic) in reducing PSQI global scores [MD = −1.17, 95% CI (−2.26, −0.08), *p* = 0.03]. However, in subgroup analysis, regardless of MA [MD = −1.12, 95% CI (−2.40, 0.16), *p* = 0.09] or EA [MD = −1.40, 95% CI (−3.24, 0.45), *p* = 0.14], acupuncture was only as effective as Western medicine in reducing PSQI global scores. Furthermore, there was no interaction effect between MA and EA (χ^2^ statistic 0.06, df = 1, *p* = 0.81). Similar results were also identified in the subgroup analyses based on either principle of acupuncture prescription or needle retention time under PSQI (Appendix 9).

##### Sensitivity Analysis

With the purpose of addressing the high heterogeneity and checking the robustness of the pooled effect size, sensitivity analysis was carried out based on the outcome of PSQI global scores to ensure the results were not due to one or two studies. We chose influence analysis, by removing one study at a time and then recalculating the combined estimate on the remaining studies to evaluate the stability of the results. We did not execute sensitivity analysis for the other outcome measures due to the small number of studies included (<10). The results implied that each single study had little impact on the pooled estimate effects of PSQI, and the overall robustness and reliability of our study results were relatively high (Appendix 10).

##### Meta-Regression Analysis

Similar to the sensitivity analysis, meta-regression analysis was only carried out based on the outcome of PSQI global scores as well. We performed univariate meta-regressions to investigate the sources of heterogeneity by treating publication year, study sample size, and acupuncture stimulation (MA or EA) as covariates. Multifactor meta-regressions were performed to find the sources of heterogeneity by taking medication used in controls (antidepressant alone, hypnotic alone, or antidepressant + hypnotic) and diagnostic criteria (ICD-10, DSM-IV, DSM-V, or CCMD-3) as covariates. However, the heterogeneity across the 10 included studies could not be substantially explained by publication year (*I*^2^ = 90.43%, τ^2^ = 2.75, *p* = 0.36), study sample size (*I*^2^ = 90.73%, τ^2^ = 3.07, *p* = 0.66), acupuncture stimulation (*I*^2^ = 91.25%, τ^2^ = 3.16, *p* = 0.91), medication used in controls (*I*^2^ = 92.78%, τ^2^ = 3.13, *p* = 0.65), or diagnostic criteria (*I*^2^ = 90.69%, τ^2^ = 2.66, *p* = 0.39; Appendix 11).

##### Acupuncture Combined With Western Medication *vs*. Western Medication

Under this category, six studies (*n* = 398) were included. All Western medications used in these six trials were antidepressants. Meta-analysis was carried out only for PSQI, HAMD, and total clinical effectiveness rate, but not for other indicators because there were fewer than three included trials for each of them. Similarly, subgroup analysis was performed where necessary.

##### Data Synthesis

###### PSQI Global Scores.

**Figure 5:**
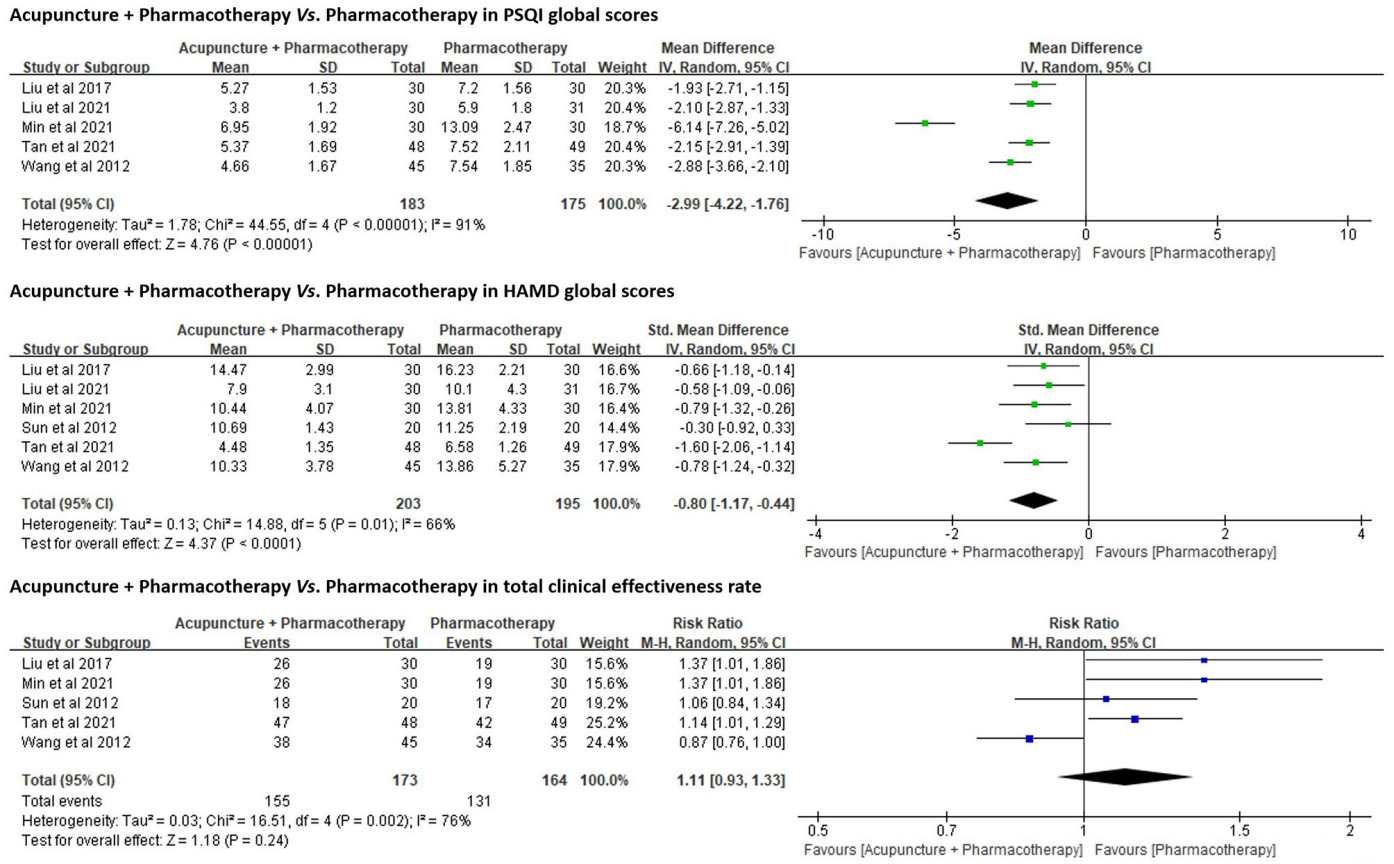
Forest plots of acupuncture + Western medication *vs*. Western medication in PSQI, HAMD, and total clinical effectiveness rate.

PSQI was adopted as an outcome in five trials (n = 358) (50, 51, 53–55). A random-effects model was used to pool data (*p* < 0.01, I^2^ = 91%). The results favored acupuncture combined with Western medication in reducing PSQI global scores [MD = −2.99, 95% CI (−4.22, −1.76), *p* < 0.01; Figure 5].

###### HAMD Global Scores.

All six trials (n = 398) used HAMD as an outcome. The results favored acupuncture in reducing HAMD global scores [SMD = −0.80, 95% CI (−1.17, −0.44), *p* < 0.01; Figure 5].

###### Total Clinical Effectiveness Rate.

Five trials (n = 337) (51–55) compared the total clinical effectiveness rates between acupuncture combined with pharmacotherapy and pharmacotherapy alone for DI (Appendix 8), while no significant differences were identified between both groups [RR = 1.11, 95% CI (0.93, 1.33), *p* = 0.24; Figure 5].

##### Subgroup Analysis

For PSQI and HAMD, there was no significant interaction effect between subgroup analysis that was prespecified: MA *vs*. EA (χ^2^ statistic 0.03, df = 1, *p* = 0.87 in PSQI; and χ^2^ statistic 0.80, df = 1, *p* = 0.37 in HAMD), fixed acupuncture prescription *vs*. semi-standardized acupuncture prescription (χ^2^ statistic 2.13, df = 1, *p* = 0.14 in PSQI; and χ^2^ statistic 0.13, df = 1, *p* = 0.72 in HAMD), and high-frequency acupuncture treatment sessions *vs*. low-frequency acupuncture treatment sessions (χ^2^ statistic 0.82, df = 1, *p* = 0.37 in PSQI; and χ^2^ statistic 1.95, df = 1, *p* = 0.16 in HAMD) (Appendix 12). We did not perform subgroup analyses based on the HAMD version or different medication in the controls because not all six studies provided version information and all medications used in the control group were antidepressants.

A meaningful discovery about total clinical effectiveness rate was identified. When all five trials were pooled for effect size, there were no significant differences between acupuncture combined with antidepressants and antidepressants alone. Yet, in subgroup analysis, the results favored MA [RR = 1.23, 95% CI (1.05, 1.43), *p* < 0.01]. There remained no differences between EA combined with antidepressants and antidepressants alone [RR = 0.94, 95% CI (0.77, 1.13), *p* = 0.51]. Furthermore, there was a significant interaction effect between MA and EA (χ^2^ statistic 4.69, df = 2, *p* = 0.03). We did not identify a significant interaction effect between different principles of acupuncture prescription (χ^2^ statistic 0.07, df = 1, *p* = 0.80) or between different acupuncture treatment frequencies (χ^2^ statistic 0.57, df = 1, *p* = 0.45) under total clinical effectiveness rate (Appendix 12).

In addition, each included trial addressed this comparison (acupuncture + Western medication *vs*. Western medication) had a 30-min needle retention time, and no subgroup analysis was required.

##### Acupuncture *vs*. Waitlist Control

No studies were identified under this comparison.

##### Acupuncture *vs*. CBT-i

No studies addressed this comparison.

#### Effect of Acupuncture on Insomnia as a Residual Symptom of Previous or Partially Remitted Depression

Two RCTs (26, 37) (*n* = 228) with a three-arm parallel design (EA *vs*. placebo-EA *vs*. sham-EA) from the same research team (Hong Kong, China) investigated the efficacy and safety of acupuncture on residual insomnia after remission or partial response of a major depressive disorder. Both RCTs presented that EA was well-tolerated but had very limited positive effects that were considered to be only non-specific effects. In the first study (published in 2011) (37), researchers found a slight advantage of EA and sham-EA over non-invasive placebo-EA in reducing PSQI and ISI scores at both 1- and 4-week post-treatment, while no significant difference was identified between EA and sham-EA at both time points. In addition, there was no significant between-group difference in actigraphy-derived objective sleep parameters (TST, SOL, SE, and WASO) as well as in HAMD and SDS scores among three acupuncture therapies. As an extension and optimization of the first study, the second study (published in 2015) (26) expanded the sample size, enriched the sample sources, and included more empirical acupoints used for insomnia in the intervention regimen. In this trial, there was a higher proportion of participants having sleep-diary-derived SOL <30 min in EA and placebo-EA groups at 1-week post-treatment, in comparison with the proportion of those in the sham-EA group, while no significant difference was identified between EA and placebo-EA. However, the researchers found no significant between-group difference among three acupuncture therapies in most of the other outcomes, including PSQI scores, actigraphy-derived sleep parameters (TST, SOL, SE, and WASO), and HAMD and SDS scores (Table 2).

### Publication Bias Test

**Figure 6:**
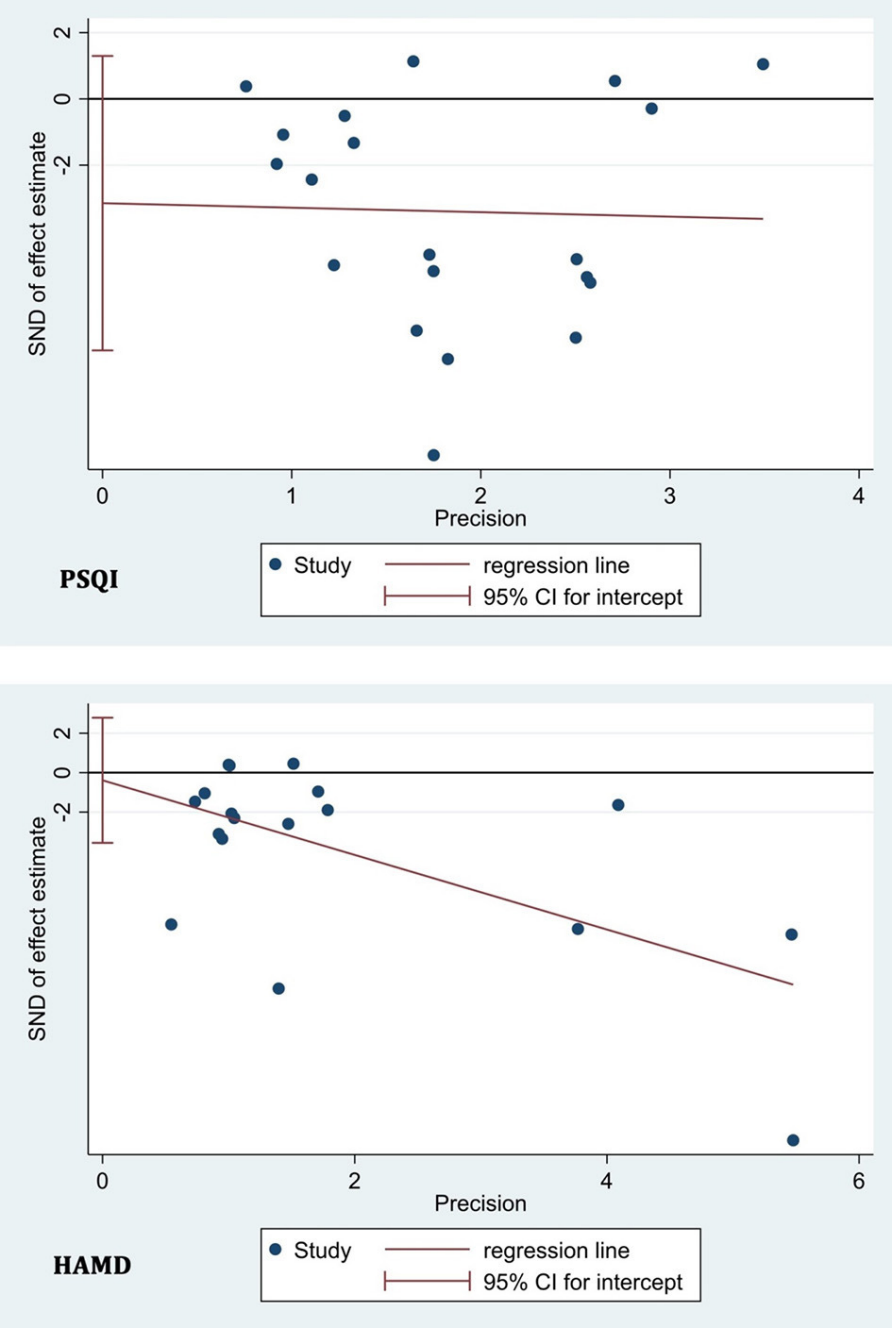
Publication bias test based on PSQI and HAMD.

We adopted a linear regression analysis (Egger's test) to detect the publication bias based on PSQI in 20 included studies and based on HAMD in 18 included studies and found no statistically significant effect (*p* = 0.154 for PSQI and *p* = 0.799 for HAMD) (Figure 6). Publication bias tests were not carried out for the other outcome measures due to the small number of studies (<10).

### Certainty and Quality of Evidence

The certainty and quality of evidence derived from meta-analyses of 15 major outcomes are illustrated in Appendix 13. In pursuance of the GRADE system, the quality of evidence ranged between very low and moderate (four were rated as “Very low;” 10 were rated as “Low;” and only one was rated as “Moderate”). The most common degradation factor was the risk of bias within the included RCTs, which involved 86.7% of outcomes.

## Discussion

### Summary of Findings

Among patients with insomnia as a major symptom in active depression (that is, depression accompanying/comorbid with insomnia), acupuncture appeared to be superior to either placebo-acupuncture or antidepressants and/or hypnotics in improving both poor sleep and depressive symptoms. Acupuncture combined with these psychotropic substances certainly showed better effects than psychotropic substances alone. The reduction of PSQI and HAMD global scores varied from 1.2 to 3.1 points and 0.5 to 2.7 points, respectively, with significant clinical relevance. Nevertheless, quality of evidence supporting these positive results was very low to moderate owing to a lack of blinding of patients and evaluators. In the treatment of patients with insomnia as a residual symptom in previous or partially remitted depression, acupuncture appeared to only possess marginally better efficacy than placebo-acupuncture and had no superior efficacy to sham-acupuncture, indicating that the observed differences between verum- and placebo-acupuncture might be due to non-specific effects of needling. This conclusion is largely reliable as the evidence was derived from two high-quality and stringently designed RCTs (26, 37). Because of inadequate data, it is premature to conclude the intermediate- and long-term benefits and risks of acupuncture for patients with DI. Also, there were no data available clarifying the efficacy differences between acupuncture and CBT-i, or whether acupuncture combined with CBT-i was superior to CBT-i alone. Acupuncture appeared to be well tolerated and safe as the AEs were only mild and far less than those reported for antidepressants and/or hypnotics. The most frequent AE of acupuncture was hand numbness and/or pain at acupoints, which usually resolved quickly after the needles were removed. Overall, acupuncture is safe in the management of DI, while its efficacy cannot be definitely concluded due to insufficient numbers and/or quality deficiencies of included RCTs.

### Strengths, Limitations, and Comparison With Previous Systematic Reviews

To the best of our current knowledge, this is the first systematic review and meta-analysis comprehensively investigating the collective evidence regarding the efficacy of acupuncture as an alternative or adjuvant management to standard care in the management of insomnia as a major or residual symptom among patients with active or previous/partially remitted depression. For patients who have no access to CBT-i or patients whose symptoms are not well-controlled under the existing Western medication schedule or patients who are reluctant to immediately give up Western medication, our review in particular provides one more option and supports augmentation of hypnotic and antidepressive effects by combined application of acupuncture and conventional regimens (antidepressant and/or hypnotic). This is consistent with previous perceptions of the add-on effect of acupuncture (56). In the medical work *Schizophrenia, Sleep, and Acupuncture*, authors recommend that patients who are on psychotropic substances can consider receiving acupuncture as a supplementary therapy, because through restoring the harmony and balance in the body, acupuncture could enhance the effect of psychotropic agents (56).

We are conscious of five existing systematic reviews and meta-analyses (four in Chinese and one in English) on a similar theme (25, 57–60). However, four of them were carried out at least 5 years ago (25, 58–60). None of these five reviews discussed insomnia as a major or residual symptom of depression separately. All these reviews included many different forms of acupoint-based therapies, such as moxibustion (57, 58), auricular acupressure (57, 58, 60), intradermal needling (57, 58), fire acupuncture (57–59), and/or even Chinese herbal/patent medicine (25, 60) and ultraviolet blood irradiation therapy (25). Furthermore, some data derived from patients with DI due to stroke (25, 57) or malignant tumor (25) or patients with comorbid DI and anxiety disorder (25, 57–59) were also included for meta-synthesis. Four out of these five systematic reviews (25, 57–59) included at least one of three apparently ill-designed RCTs (61–63) in which diverse types of antidepressants were used as controls and the number of participants using each type of antidepressant was unclear. All such aforementioned practice introduces extra variability and makes it difficult to interpret the results. Here, we only focused on common forms of acupuncture (MA or EA) and ruled out RCTs in which patients were diagnosed as DI due to or comorbid with other physical and mental disorders, to reduce variability and subsequently to better reflect the real effect size. It is also noteworthy that incomplete retrieval was identified as a common issue in previous reviews. For instance, two RCTs (44, 52), published in 2012 and 2013, that were included in our review were not included in the four previous systematic reviews (25, 58–60), even though they met the inclusion criteria of those reviews. Those four systematic reviews performed literature search work no earlier than 2016. This thus can be seen as a case of incomplete search. Finally, all these five reviews did not consider or mention the different versions of HAMD employed in the included RCTs. In evidence synthesis, MD but not SMD thereby was inappropriately adopted for pooling the estimated effect size, which also undermined the reliability and accuracy of their results.

In addition to the stricter selection criteria and usage of widely accepted analysis tools as mentioned above, the merits of our current review also included the following: (1) considering the distinct clinical characteristics between two types of DI, the therapeutic effect of acupuncture on them was separately analyzed and different conclusions were drawn; (2) with limited data synthesis (derived from three RCTs), we were the first to report the significant efficacy differences between verum- and placebo-acupuncture on insomnia as a major symptom among patients with depression; (3) the STRICTA checklist and the GRADE system were introduced to appraise the reporting quality and evidence quality, respectively; and (4) we provided a complete list of excluded studies with justification for the exclusion of each, so as to present a transparent and comprehensive screening process and enable readers to determine whether there is a risk of unjustified exclusion (64). The strengths of our study enable a more solid and reliable reference for clinicians when translating evidence into clinical practice for managing different types of DI.

The present review has a few limitations, the first of which was the number of studies available for assessment and the small sample sizes of each. Secondly, both the overall quality of the included trials and the quality of the synthesized evidence arising from the data of these trials was less than satisfactory. Thirdly, heterogeneity was high across the studies. We adopted sensitivity, subgroup, and meta-regression analyses but were unable to reasonably explain the potential source of the heterogeneity in specificity. Fourthly, acupuncture is a complex intervention, and the skills and experience of the operator are the linchpin. Some included studies did not clearly describe acupuncture parameters including depth of insertion and/or needle type used, and only three studies (26, 27, 37) explained the background of the acupuncturist. Those limitations impact the reproducibility and assessment of the real contribution. Fifthly, the medium- to long-term efficacy of acupuncture on DI remains elusive as only five studies (26, 27, 37, 42, 46) included follow-up. Sixthly, we excluded DI in or following stroke, cancer survivors, chronic pain, and feminine-specific physiological stage from the literature screening phase, although these conditions are quite common and the symptom cluster of sleep and emotional dysregulation in these special populations could be more severe (19). Caution thereby should be exercised in generalizing the positive benefits of the current review to DI in these special populations as mentioned. Finally, all RCTs were carried out in China. The sociocultural context may interact with biologic mechanisms and mediate the patient's experience of acupuncture (65). Due to cultural identity and confidence, Chinese individuals usually have high expectations of acupuncture (originating in China), which may inadvertently inflate and exaggerate the treatment outcomes (66). It is therefore unpredictable whether the results could be replicated in communities of other races and cultures. Further rigorous and well-designed trials with larger and more diverse clinical samples are required to build powerful and conclusive evidence. The improvement in reporting quality (completeness and clarity) of the acupuncture scheme is also required, which will be conducive to maximizing the transparency, reproducibility, and standardization of the treatment procedure as well as to facilitating the utilization of this therapy by clinical practitioners.

### Interpretation of Findings

Since ancient times, acupuncture has been used to treat insomnia and/or depression (67, 68). In the past decade, at least 31 systematic reviews and at least 34 systematic reviews investigating the treatment of depression (67) and insomnia (68), respectively, with acupuncture, have been published, reflecting the continuous growth of attention and research interest in the management of psychological and sleep disorders by using acupuncture in the international medical community. Unfortunately, design deficiencies of included original RCTs or heterogeneities across individual studies hinder these reviews from yielding high-quality results and drawing firm conclusions (67, 68). Common shortcomings with potential solutions can be referred to in the findings outlined in these two overviews (67, 68), and our review has some additional reminders for clinical practitioners and researchers. In exploring the efficacy of acupuncture, four studies employed selective serotonin reuptake inhibitors (SSRIs), such as paroxetine (42), sertraline (41), escitalopram (43), and citalopram (44), alone as positive controls. The usage (particularly short-term usage) of this class of antidepressive agents however may deteriorate sleep quality, particularly increasing REM-SOL (5) and disrupting sleep continuity (5, 8). Reported by the US Food and Drug Administration, the average prevalence of treatment-emergent insomnia and daytime somnolence was 17% and 16%, respectively, among depressed patients treated with SSRIs (8, 69). In future studies with the same design (acupuncture *vs*. Western medicine), agomelatine, mirtazapine, or trazodone might be more appropriately chosen as valid controls, thus providing a truer picture of the efficacy of acupuncture and enhancing the trustworthiness of the results. Nearly three quarters of individuals suffering from depression will relapse at some point in their lives (5); insomnia not only affects a large proportion of the population on a recurrent and tenacious basis (70) but also can contribute to the depression relapses (5, 71). Hence, the long-term effect of any therapeutic strategy in the management of DI is of significant clinical importance. Insufficient data supported effect-size synthesis in this review and prevented us from judging how acupuncture treatment benefits were maintained at follow-ups. Future RCTs are recommended to include rational intermediate- and long-term follow-ups. Only three trials (26, 27, 37) included dropout/withdrawal cases in the final analysis. Other trials directly excluded data from these cases, which might partially skew the reliability of results. Such defects should also be addressed in future studies through sound statistical approaches (e.g., last observation carried forward and multiple imputation), so that an unbiased determination of the efficacy of acupuncture can be acquired.

In spite of these restrictions, the current review contributes many valuable new information and empirical insights.

A reduction of 3 points or more in PSQI global scores (72) and HAMD global scores (73) was chosen to indicate a minimal clinically significant difference in sleep and depression symptoms, respectively. A majority of the studies we retrieved concentrate on insomnia as a major symptom of active depression. Within this category of patients, our review found that acupuncture was better than either placebo-acupuncture or standard care (antidepressant and/or hypnotic) in reducing PSQI scores by 1.2–3.1, which is of clinical importance. Thus, acupuncture is significantly better than placebo and at least equivalent to standard care in improving poor sleep among patients with depression. Meanwhile, for the depressive symptoms in these patients, acupuncture was better than either placebo-acupuncture or standard care in reducing HAMD scores by 0.5–2.7. Although this score did not reach 3 points, which represented “minimal improvement,” it suggests that acupuncture may have an antidepressive effect that is at least no worse than that of antidepressants and/or hypnotics. In the further subgroup analysis, we identified that in comparison with standard pharmacotherapy, acupuncture with high-frequency treatment sessions (≥5 sessions per week) was more effective in improving patients' sleep quality and quantity but was not more effective in improving patients' depressive symptoms. Nevertheless, there was no significant difference between acupuncture with low-frequency treatment sessions (<5 sessions per week) and standard care in improving both insomnia and depression symptoms among patients. In addition, the stimulation of acupuncture (EA or MA), variation in treatment protocol (standardized treatment protocol with fixed acupoints or semi-standardized treatment protocol with predefined acupoints in combination with acupoints selected based on symptoms and/or TCM patterns), and needle retention time (20–30 or 30 min) did not appear to have a significant impact on the aforementioned results. On the basis of these findings, we here recommend that, when delivering acupuncture treatment for patients with active depression and concomitant insomnia, clinical practitioners may consider increasing the frequency of acupuncture treatment, such as providing five treatment sessions per week, to enhance the hypnotic effect of acupuncture. As a single non-drug therapy, acupuncture is at least as effective as psychotropic medications. Its effect in concurrently ameliorating both depression and insomnia and its safety are of significant value to patients with depression who also have concerns over their liver or kidney function, which could be compromised by pharmacotherapy. Despite the absence of correlational analysis, the trends in the change of PSQI and HAMD scores were largely positively correlated (Table 2), suggesting that as insomnia or depression ameliorated, other symptoms ameliorated as well. These findings are also consistent with the erstwhile knowledge of the complex interaction and bidirectionality between insomnia and depression (5, 74).

Insomnia is the most common residual symptom of depression (8, 75), which is not only linked to poorer quality of life (76) but also regarded as a critical factor in subsequent depression relapse (75, 76). Although the search was comprehensive, only two trials from the same research team concerning acupuncture for depression-associated residual insomnia were identified. One single-center trial (*n* = 78) (37) found a greater improvement in self-report sleep but not in objective sleep parameters or depressive symptoms, with EA being superior to non-invasive placebo-EA (Streitberger needle), but there was no difference between EA and sham-EA (superficial needling at non-acupoints). The team then ran a multicenter trial (26) (*n* = 150) and found similar results. The authors concluded that the limited hypnotic effect of acupuncture was largely due to its non-specific effects, such as general physiological effects of needling and electrostimulation (26, 37). These two RCTs are well-designed and graded as high-quality studies as assessed with the modified Jadad system and the Cochrane tool. We cannot deny the placebo effect of acupuncture; indeed, in the management of insomnia, positive beliefs, anticipation, and expectations concerning CAM may result in more favorable outcomes and higher satisfaction with CAM, i.e., the placebo effect (19, 77). The only concern of those two trials (26, 37) is if the acupuncture treatment was sufficient. The treatment was provided three times per week for 3 weeks with a total of nine sessions, which is substantially fewer in comparison to a total of 12–42 sessions over 4–24 weeks in trials included under the category addressing insomnia in active depression. This is consistent with our findings in a previous systematic review (22), which found that a minimum of 12 sessions of acupuncture treatment was needed for primary insomnia to produce a significant improvement on both TST and SE, and thus, it could be considered the “lowest threshold dosage” (22). Also, there may be a positive dose–response relationship between acupuncture's hypnotic effect and its dose (22). Dosage is a crucial factor for acupuncture's optimal clinical efficacy and has been found as one of the main reasons for the failure of many acupuncture trials to yield positive results (78). The robustness of the results of those two trials (26, 37) should be retested in future studies by further increasing the acupuncture dosage or after identifying the optimal acupuncture dosage (e.g., frequency of sessions, needle retention time, and/or length of treatment period). Despite satisfactory effects in attenuating residual insomnia of previous or partially remitted depression, conventional hypnotics, particularly with high doses, have been associated with antidepressant refractoriness and/or an elevated risk of developing a psychiatric disorder (76). Therefore, other potentially effective therapies (including combined application of acupuncture and hypnotic/antidepressant/CBT-i/repetitive transcranial magnetic stimulation) also need to be actively sought and determined; after all, the available evidence does not, in any case, support the use of acupuncture alone in managing residual insomnia associated with previous or partially remitted depression.

The third purpose of this systematic review was to determine whether acupuncture could amplify the therapeutic effect and/or attenuate the adverse reactions caused by psychotropic substances. Only limited evidence was attainable. Six RCTs (50–55) under this category showed that the combined therapy was more effective than antidepressants (venlafaxine, mirtazapine, or paroxetine) alone in ameliorating both poor sleep and depressed mood among patients with active depression accompanying or comorbid with insomnia. Moreover, subgroup analysis based on different acupuncture methods implied that MA might produce better efficacy than EA when combined with antidepressants. This superiority of MA over EA may be explained by the major demerit of EA, that is, the development of adaptation or tolerance that arises in fixed pulses with settled intensity and frequency (79). Two (52, 55) of the six RCTs reported that AEs in acupuncture combined with venlafaxine or paroxetine were slightly less than those in venlafaxine (5/20 *vs*. 6/20) or paroxetine (6/30 *vs*. 7/30) alone. Another two (51, 53) RCTs employed the Asberg Side Effects Rating Scale (SERS) and found that SERS scores were significantly lower in the combined therapy, indicating that AEs caused by mirtazapine or paroxetine were diminished when acupuncture was superimposed. Unfortunately, all these positive findings might be challenged because of the inadequate sample size and the less-than-rigorous design in original RCTs. Also in need of note here is that we tried but ultimately were not able to access the SERS from either the reference lists or the corresponding authors of these two papers (51, 53). The reliability of SERS hence could not be judged. We recommend the inclusion of internationally endorsed and standard tools such as the Antidepressant Side-Effect Checklist (80), the UKU Side Effects Rating Scale (81), and/or the Frequency, Intensity, and Burden of Side Effects Rating Scale (82) in future studies to truly reflect the real impact of acupuncture on antidepressant-induced AEs. A previous systematic review revealed that acupuncture was helpful in reducing antidepressant-induced side reactions in the first 6 weeks of the treatment period among patients with depression (79). Most RCTs included in this review did not provide data on AEs, preventing evidence synthesis. Hence, whether acupuncture produces a similar benefit of attenuating side effects caused by antidepressants and/or hypnotics during the early onset of DI would be an attractive direction for future investigation.

Acupoint selection is one of the linchpins affecting the clinical effectiveness of acupuncture treatment (83). In the 19 trials focusing on insomnia as a concomitant symptom in active depression, the three most frequently utilized acupoints were GV20, EX-HN3, and HT7 (Table 1). Depending on “Indications of Acupuncture Points [GB/T 30233-2013]” (National Standard of the People's Republic of China, 2013 version) (84), these acupoints are all classic acupoints for the treatment of psychiatric and psychological disorders. The hypnotic and antidepressant effects possessed by these three acupoints can not only be explained through TCM meridian theory (84) but have also been confirmed by modern medicine approaches. In rodents with sleep deprivation, the instant sedative effect produced by acupuncture at GV20 is achieved by reducing the excitability of the cerebral cortex (85). This stimulation may also be associated with the regulation of 5-hydroxyindoleacetic acid and enhancement of acetylcholinesterase activity in the brain (85). In depressed rodents, acupuncture at GV20 and EX-HN3 showed significant antidepressive effects that were not inferior to those of fluoxetine, and this effect was associated with acupuncture-induced inhibition of the NLRP3 inflammasome signal pathway in the prefrontal cortex and reduction of cerebral inflammation (86). Similarly, acupuncture at HT7 was found to activate cerebral regions highly associated with cognition, sleep, and emotion (70). Coincidentally, in a previous systematic review summarizing herb/acupuncture prescriptions for insomnia, these three acupoints were also identified to be frequently used but were non-specific for TCM patterns (87). We thereby recommend that in the future treatment of active depression with insomnia as a concomitant symptom, practitioners can consider including these three acupoints as the core prescription and other acupoints that are appropriate to patients' different TCM syndromes, in order to comply with the individualized, syndrome differentiation principle of TCM. Since acupuncture has not been proven to be effective for residual insomnia associated with previous or partially remitted depression, we do not recommend any acupoints for this type of insomnia.

Six RCTs (26, 27, 37, 39, 44, 52) reported objective sleep parameters in participants, five of which also reported changes in PSQI. Trends between subjective and objective outcomes were largely consistent, albeit no correlation analysis was executed (Table 2). When patients spontaneously reported an improved sleep (reflected by decreased PSQI scores) following acupuncture treatment, researchers usually saw a shortened SOL (39), prolonged TST (27, 39, 44), increased SE (27, 39), and/or reduced WASO (39) in PSG/actigraphy data. The major difference between the subjective and objective indices was the number of awakenings. Two trials (27, 44) demonstrated that acupuncture significantly reduced ATs, while another trial (39) exhibited that acupuncture reduced only WASO but not ATs. No data synthesis was performed due to the heterogeneous study design. In any case, most of the findings are encouraging. Sleep in depression is characterized by disturbances of sleep continuity, including early morning awakening, prolonged SOL, decreased SE, and increased WASO and ATs (8, 88). The available findings at least confirm that the latter three types of disrupted sleep continuity may benefit from acupuncture treatment. Only two included RCTs (39, 52) investigated the effects of acupuncture on sleep structure/architecture through PSG and provided very limited evidence. One study revealed that acupuncture significantly shortened REM-SOL (39). But indeed, alteration of REM sleep (e.g., reduced REM-SOL, prolonged REM duration and first REM period, and increased REM density) itself is the most prominent feature of the sleep structure/architecture among depressed patients (8). It is hence indeterminate whether the observed change in REM-SOL was a manifestation of the development of depression over time or whether it was caused by acupuncture. Worse still, the relative excess of REM appears to come at the expense of stage N3 sleep (SWS) (89). Another study (52) illustrated that either acupuncture combined with venlafaxine or venlafaxine alone reduced REM%, increased NREM%, and prolonged TST, and the latter two effects caused by the combined therapy were more significant. These findings came as a surprise, because a reduction of SWS and disinhibition of REM sleep are typical characteristics of sleep in depressed patients (8), and this altered sleep structure/architecture appears to be normalized by acupuncture. Another electroencephalogram characteristic of depressed patients is a decreased delta ratio (the ratio of SWS between delta wave activity in the first and second sleep cycles) (8). The effect of acupuncture on this parameter, as well as the aforementioned REM sleep duration and density, should be further investigated in future studies.

The particular mechanism by which acupuncture affects DI is intriguing. Some evidence indicates that impaired metabolism of plasma neuropeptide Y (NPY) and the decreased plasma NPY may be involved in the pathogenesis or pathophysiological process of depression (90) and insomnia (91). Increased serum substance P (SP) has been observed in a proportion of patients with depression (92). Another study from China observed dysfunction in peripheral NPY- and SP-ergic neurons in patients with either primary insomnia or DI, with the latter group showing more severe SP-ergic neuronal dysfunction (93). One trial in the current review reported significant improvements in both insomnia and depressive symptoms among patients with DI after receiving acupuncture treatment, accompanied by increased serum NPY and decreased SP levels, and these positive changes were not identified in the placebo-acupuncture group (39). Another trial showed that abnormalities in T-lymphocyte subsets and immunoglobulin due to DI were normalized by acupuncture intervention (45). Additionally, increased inflammatory cytokines levels (e.g., interleukin-6 and tumor necrosis factor) (5), dysregulation of neurotransmitters (e.g., serotonin and norepinephrine) (5), or gut microbiota alterations (94) may also explain the bidirectional relationship between depression and insomnia and/or may be involved in the pathogenesis and development of DI. Whether acupuncture's impact on DI is also associated with its modulation of these cellular events deserves further exploration and elucidation in future studies. Existing hypotheses also include that acupuncture exerts a hypnotic effect through the modulation of regional brain activity and functional connectivity, particularly in emotion-related areas (95). Shedding light on the mechanisms underlying acupuncture for DI *via* imaging techniques, e.g., functional magnetic resonance imaging, diffusion tensor imaging, and magnetic resonance spectroscopy, thereby would be another valuable and interesting direction.

## Conclusions

This review has provided a low to moderate level of evidence supporting acupuncture as a safe and effective remedy alternative to or adjuvant to conventional pharmacotherapy (antidepressant and/or hypnotic) in improving insomnia as a major symptom among patients with active depression as well as their depressed mood. Furthermore, the complaint of disrupted sleep continuity in this category of patients is most likely to benefit from acupuncture. Future studies need to include appropriate patient–evaluator blinding methods in the trial design, to observe the intermediate- and long-term effects of acupuncture, to investigate whether there is a synergistic effect between acupuncture and CBT-i, and to introduce PSG as an outcome metric to elucidate the mechanisms underlying acupuncture on sleep architecture/structure, particularly REM-SOL, REM%, and NREM%. The effect of acupuncture on residual insomnia associated with previous or partially remitted depression requires further research employing optimal dosage of treatment. Searching for other potentially positive therapeutic strategies, including acupuncture combined with standard care (antidepressant and/or hypnotic, and/or CBT-i) in managing this type of insomnia, also remains warranted and urgent.

## Data Availability Statement

The original contributions presented in the study are included in the article/Supplementary Material, further inquiries can be directed to the corresponding author/s.

## Author Contributions

ZZ, GK, and Q-QF designed this review. W-JZ, Q-QF, and F-YZ performed the database search, data extraction, and statistical analyses. F-YZ, QQ-F, and ZZ were involved in the quality assessment and bias risk analysis. F-YZ drafted the manuscript. SS, RC, and ZZ provided critical comments for revising the manuscript. All authors contributed to the article and approved the submitted version.

## Funding

This work was sponsored by RMIT Research Stipend Scholarship, RMIT University, Australia, and University's scientific research project, Shanghai Sanda University (2021zz02-yj) to F-YZ and the National key R&D projects in the 14th five year plan (2021YFC2501500) to W-JZ.

## Conflict of Interest

The authors declare that the research was conducted in the absence of any commercial or financial relationships that could be construed as a potential conflict of interest.

## Publisher's Note

All claims expressed in this article are solely those of the authors and do not necessarily represent those of their affiliated organizations, or those of the publisher, the editors and the reviewers. Any product that may be evaluated in this article, or claim that may be made by its manufacturer, is not guaranteed or endorsed by the publisher.
