# Supplementary material for: The Role of Acupuncture in the Management of Insomnia as a Major or Residual Symptom Among Patients With Active or Previous Depression: A Systematic Review and Meta-Analysis
Source: Front Psychiatry. 2022 Apr 15;13:863134. doi: 10.3389/fpsyt.2022.863134 (PMC9051249; doi:10.3389/fpsyt.2022.863134)
Supplement: Supplementary file 1 [file Data_Sheet_1.pdf]

Appendicies

Appendix 1 Search strategy for each database

Databases	Search strategy
MEDLINE (via PubMed)	#1 ((randomized controlled trial[Publication Type]) OR (controlled clinical trial[Publication Type]) OR (randomized[Title/Abstract]) OR (placebo[Title/Abstract]) OR (randomly[Title/Abstract]) OR (trial[Title]) OR ("Clinical Trials as Topic"[Mesh:noexp])) NOT ((animals[MeSH Terms]) NOT humans[MeSH Terms]) - Saved search<br>#2 acupuncture[MeSH Terms]<br>#3 acupuncture[Title/Abstract]<br>#4 manual acupuncture[Title/Abstract]<br>#5 electropuncture[Title/Abstract]<br>#6 acupoint[Title/Abstract]<br>#7 #2 or #3 or #4 or #5 or #6<br>#8 depression[MeSH Terms]<br>#9 depressive disorder[Title/Abstract]<br>#10 depression[Title/Abstract]<br>#11 #8 or #9 or #10<br>#12 insomnia[MeSH Terms]<br>#13 insomnia[Title/Abstract]<br>#14 "sleep Initiation and maintenance disorders"[Title/Abstract]<br>#15 sleep disorder[Title/Abstract]<br>#16 dyssomnias[Title/Abstract]<br>#17 sleep wake disorders[Title/Abstract]<br>#18 #12 or #13 or #14 or #15 or #16 or #17<br>#19 #1 and #7 and #11 and #18
Cochrane Central Register of Controlled Trials (CENTRAL)	#1 MeSH descriptor: [Acupuncture] explode all trees<br>#2 ("needle"):ti,ab,kw<br>#3 (acupuncture):ti,ab,kw<br>#4 (manual acupuncture):ti,ab,kw<br>#5 (electropuncture):ti,ab,kw<br>#6 (acupoint):ti,ab,kw<br>#7 #1 or #2 or #3 or #4 or #5 or #6<br>#8 MeSH descriptor: [Depression] explode all trees<br>#9 (depression):ti,ab,kw<br>#10 (depressive disorder):ti,ab,kw<br>#11 #8 or #9 or #10<br>#12 MeSH descriptor: [Depression] explode all trees<br>#13 (insomnia):ti,ab,kw<br>#14 ("sleep Initiation and maintenance disorders"):ti,ab,kw<br>#15 (sleep disorder):ti,ab,kw<br>#16 (sleep wake disorders):ti,ab,kw<br>#17 (dyssomnias):ti,ab,kw<br>#18 (sleep disorder):ti,ab,kw

	#19 #12 or #13 or #14 or #15 or #16 or #17 or #18<br>#20 #7 and #11 and #19
EMBASE	#1 'crossover procedure':de OR 'double-blind procedure':de OR 'randomized controlled trial':de OR 'single-blind procedure':de OR random*:de,ab,ti OR factorial*:de,ab,ti OR crossover*:de,ab,ti OR ((cross NEXT/1 over*):de,ab,ti) OR placebo*:de,ab,ti OR ((doubl* NEAR/1 blind*):de,ab,ti) OR ((singl* NEAR/1 blind*):de,ab,ti) OR assign*:de,ab,ti OR allocat*:de,ab,ti OR volunteer*:de,ab,ti<br>#2 'needle'/exp OR needle<br>#3 'acupuncture'/exp OR acupuncture<br>#4 'manual acupuncture':ab,ti<br>#5 'electropuncture':ab,ti<br>#6 'acupoint':ab,ti<br>#7 'acupuncture':ab,ti<br>#8 #2 OR #3 OR #4 OR #5 OR #6 OR #7<br>#9 'depression'/exp<br>#10 'depression':ab,ti<br>#11 'depressive disorder':ab,ti<br>#12 #9 OR #10 OR #11<br>#13 'insomnia'/exp<br>#14 'sleep initiation and maintenance disorders':ab,ti<br>#15 'sleep disorder':ab,ti<br>#16 'sleep wake disorders':ab,ti<br>#17 'dyssomnias':ab,ti<br>#18 'sleep disorder':ab,ti<br>#19 #13 OR #14 OR #15 OR #16 OR #17 OR #18<br>#20 #1 AND #8 AND #12 AND #19
China National Knowledge Infrastructure (CNKI)	(篇名=抑郁) AND (篇名=失眠) OR (篇名：睡眠障碍) AND (篇名：针灸) OR (篇名：针刺) OR (篇名：电针) OR (篇名：针) OR (篇名：手捻针) OR (篇名：穴) AND (主题：随机对照试验)
Wanfang database	题名或关键词:("抑郁") and 题名或关键词:("失眠") or 题名或关键词:(睡眠障碍) and 题名或关键词:(针灸) or 题名或关键词:(穴) and 主题:(随机对照试验)
Chongqing VIP database (CQVIP)	题名或关键词=抑郁+抑郁症状+抑郁情绪 AND 题名或关键词=失眠+sleeplessness+不寐 AND 题名或关键词=针灸+acupuncture+电针 OR 题名或关键词=针刺+针灸+针法 AND 任意字段=随机对照试验
China biomedical literature service system (SinoMed)	"抑郁"[标题:智能] AND "失眠"[标题:智能] OR "睡眠障碍"[标题:智能] AND "针灸"[标题:智能] OR "针"[标题:智能] OR "针刺"[标题:智能] OR "电针"[标题:智能] OR "穴"[标题:智能] AND "随机对照试验"[常用字段:智能]
US ClinicalTrials.gov	(depression OR depressive disorder) AND (insomnia sleep OR Initiation and maintenance disorders OR sleep disorder OR sleep wake disorders OR dyssomnias OR sleep disorder) AND (acupuncture OR manual acupuncture OR electropuncture OR acupoint OR needle)
WHO International clinical trials registry platform search portal	(depression OR depressive disorder) AND (insomnia sleep OR Initiation and maintenance disorders OR sleep disorder OR sleep wake disorders OR dyssomnias OR sleep disorder) AND (acupuncture OR manual acupuncture OR electropuncture OR acupoint OR needle)

Appendix 2 Detailed information for excluded studies

References	Reasons for exclusion
An HX. Clinical effect analysis of acupuncture in the treatment of insomnia combined with depressive disorder [article in Chinese]. *China Health Visi*on, 2021; 7:100.	no specific data for PSQI and HAMD
Cai CY. Acupuncture for depression-related insomnia based on "Treatment from *Heart* and *Gall bladder*" theory [article in Chinese]. *Master thesis*, 2014; Guangzhou University of Chinese Medicine.	control group included acupuncture
Cao JC. Acupuncture with "Soothing *Liver* and Regulating Spirit" method versus placebo-acupuncture for depression-related insomnia: A controlled trial [article in Chinese]. *Master thesis*, 2015; Guangzhou University of Chinese Medicine.	treatment and control group included needle-embedding therapy
Chen JY. Acupuncture integration protocol versus Paroxetine in the treatment of mild to moderate depression-related insomnia [article in Chinese]. *Master thesis*, 2016; Guangzhou University of Chinese Medicine.	treatment group included moxibustion
Chen L. Superficial acupuncture on the dorsal acupoints of the *Bladder Channel of Foot-Taiyang* in treating depression-related insomnia [article in Chinese]. *Master thesis*, 2021; Heilongjiang University of Chinese Medicine.	control group included acupuncture
Chen XM. Application and efficacy analysis of acupuncture in the treatment of depression-related insomnia [article in Chinese]. *Chin J Mod Drug Appl*, 2018; 12(5):70-71.	no diagnostic criteria for depression
Cheng WP, Quan S, Li XY, Zou QB, Song Y. Clinical research on strengthening squareneeding of Baihui to treat depression-related insomnia [article in Chinese]. *Chinese Archives of Traditional Chinese Medicine*, 2011; 29(12):2608-2609.	control group included acupuncture
Chung KF, Yeung WF, Zhang SP, Zhang ZJ, Wong MT, Lee WK, Chan KW. Acupuncture for persistent insomnia associated with major depressive disorder: a randomised controlled trial. *Hong Kong Med J*, 2016; 22 Suppl 2:S9-14.	duplicates [26]
Ding QG, Liu Y. Combination of acupuncture, medicine and psychological counseling in the treatment of 30 cases patients with depression-induced insomnia [article in Chinese]. *Chinese Medicine Modern Distance Education of China*, 2017; 15(17):83-85.	treatment group included Chinese herbal medicine
Ding WL. Application and efficacy analysis of acupuncture in the treatment of depression-related insomnia [article in Chinese]. *Chin Cont Med Edu*, 2018; 10(26): 155-157.	no diagnostic criteria for depression
Duan B, Guo B, Wang SJ. 50 cases of insomnia due to depression treated with electroacupuncture and Fluoxetine [article in Chinese]. *Chinese Journal of Trauma and Disability Medicine*, 2013; (2):96.	control group included acupuncture
Fu L. Clinical efficacy of low-frequency pulsed electrical acupoint stimulation on depression-related insomnia [article in Chinese]. *Master thesis*, 2016; Hubei	treatment- and control- group included auricular acupressure

University Of Traditional Chinese Medicine.	
Gao XY. Effect of acupuncture on sleep quality in patients with mild to moderate depression [article in Chinese]. *China Health Food*, 2021; (7):46-47.	no diagnostic criteria for depression
Hao L. Clinical observation of acupuncture with Trazodone in the treatment of depression-related insomnia [article in Chinese]. *World Latest Medicine Information*, 2020; 20(72):165-166.	no validated outcome indicators (sleep scales/questionnaires)
Hong YB. Clinical study of electroacupuncture on the treatment of insomnia combined with depressive disorder [article in Chinese]. *Master thesis*, 2003; Beijing University of Chinese Medicine.	no diagnostic criteria for depression
Hu RX. Acupuncture with twirling reinforcing method for depression-related insomnia with the *Heart* and *Spleen* deficiency pattern [article in Chinese]. *Master thesis*, 2017; Xinjiang Medical University.	control group included acupuncture
Xun A. Clinical observation on the treatment of depression-related insomnia by mind-tranquilizing acupuncture [article in Chinese]. *Master thesis*, 2018; Changchun University of Chinese Medicine.	control group included acupuncture
Jiang XF. Clinical efficacy analysis of acupuncture in the treatment of insomnia accompanying with depressive disorder [article in Chinese]. *Women's Health Research*, 2020; 23:118-119.	no diagnostic criteria for depression
Kuang HF. Clinical study of acupuncture combined with medication in the treatment of depression with sleep disorder [article in Chinese]. *Doctorate thesis*, 2009; Nanjing University of Chinese Medicine.	treatment group included moxibustion
Li L, Zhang HB, Niu WM, Wang YY. The efficacy of acupuncture with psychological counselling in the treatment of depression with sleep disorder [article in Chinese]. *Journal of Sichuan Traditional Chinese Medicine*, 2016; 34(6):207-210.	control group included acupuncture
Li MY. Clinical study on the improvement of sleep quality in patients with comorbid depression and insomnia by acupuncture with "Soothing *Liver* and Regulating Spirit" method [article in Chinese]. *Master thesis*, 2016; Guangzhou University of Chinese Medicine.	treatment group included intradermal needle
Li QQ. Huang GL, Feng SL. Clinical observation of acupuncture combined with ear-acupoint therapy on depression-related insomnia with *Heart-Spleen* deficiency pattern [article in Chinese]. *Journal of Traditional Chinese Medicine University of Hunan*, 2018; 38(3):302-306.	control group included acupuncture
Li QY, Dong YF. 26 cases of severe depression-related insomnia treated with electroacupuncture and Western medicine [article in Chinese]. *Chin J Inf Tradit Chin Med*, 2005; 12(3):71-72.	no validated outcome indicators (depression- and sleep-scales/questionnaires)
Li R, Liu JL, Xin YR, Li HY, Song HL, Zhang YX, Zhao CJ. Clinical observation of body acupuncture combined with wrist-ankle acupuncture in the treatment of depression-related insomnia [article in Chinese]. *China Health Care & Nutrition*, 2018; 28(34):95-96.	control group included acupuncture
Liang ML, Peng Q. Clinical observation of acupuncture in the treatment of depression-related insomnia [article in Chinese]. *Medical Information*, 2017; 30(13):127-128.	no diagnostic criteria for depression

Liu J. Comparative clinical study of acupuncture with "Soothing *Liver* and Regulating Spirit" method and non-acupoints stimulation for the treatment of depression-related insomnia [article in Chinese]. *Master thesis*, 2020; Guangzhou University of Chinese Medicine.	treatment group included intradermal needle therapy
Liu WH, Zhao CY, Lun X, Yu J. 30 cases of depression-related insomnia treated with "Sleeping Three Needles" [article in Chinese]. *Journal of Clinical Acupuncture and Moxibustion*, 2009; 25(04):5-6.	no diagnostic criteria for depression
Liu Y, Chen L. Superficial acupuncture of the dorsal acupoints of the *Bladder Channel of Foot-Taiyang* based on the theory of acupuncture-induced *Qi* regulation in treating depression-related insomnia [article in Chinese]. *Hebei Journal of Traditional Chinese Medicine*, 2020; 42(08):1233-1236.	control group included acupuncture
Liu Y. Integrated acupuncture and moxibustion program for depression-related insomnia [article in Chinese]. *Doctorate thesis*, 2017; Guangzhou University of Chinese Medicine.	treatment group included intradermal needle therapy and/or moxibustion
Luo D, Wu YN, Cai L, Li MY, Duan Q, Ma R, Wang L, Fu WB. Clinical effects of acupuncture with "Soothing *Liver* and Regulating Spirit" method on depression-related insomnia [article in Chinese]. *Chinese Journal of Gerontology*, 2017; 37(15):3837-3839.	treatment and control group included intradermal needle therapy
Luo WZ. Acupuncture with "Relieving Depression and Regulating Spirit" method on insomnia accompanying depression [article in Chinese]. *Doctorate thesis*, 2006; Guangzhou University of Chinese Medicine.	no diagnostic criteria for depression
Luo WZ, Zhang QZ, Lai XS. Effect of acupuncture treatment of relieving depression and regulating mind on insomnia accompanied with depressive disorders [article in Chinese]. *Chin Acup Moxib*, 2010; 30(11):899-903.	no diagnostic criteria for depression
Luo RG, Wang J. Clinical observation and systematic review of the effect of acupuncture in treating patients with insomnia accompanying with depressive disorder [article in Chinese]. *China Health Care & Nutrition*, 2018; 28(14):56-57.	non-standard diagnostic criteria for depression
Qu YZ. Acupuncture with "intercourse of the dragon and the tiger" method for the treatment of depression in patients with insomnia in *Liver*-depression and *Qi*-stagnation pattern [article in Chinese]. *Master thesis*, 2011; Chengdu University of TCM.	control group included acupuncture
Ren JN. Clinical observation of body acupuncture combined with wrist-ankle acupuncture in the treatment of depression-related insomnia [article in Chinese]. *Shanghai J Acup Moxib*, 2011; 30(8):527-528.	control group included acupuncture
Shen J, Zhang Y. Clinical study on depression-related insomnia by acupuncture with "Soothing *Liver* and Regulating Spirit" method [article in Chinese]. *Journal of Sichuan Traditional Chinese Medicine*, 2017; 35(10):181-183.	Western medication used in the control- and treatment- groups were unclear
Song Q. 56 cases of depression-related insomnia treated with acupuncture at GV20 and EX-HN1 [article in Chinese]. *Capital Medicine*, 2007; 14(18):48-49.	no diagnostic criteria for depression
Song SC, Lu Z, Chen H, Wang LC, Zhao JW [article in Chinese]. *Chinese Journal of Integrative Medicine on Cardio-/Cerebrovascuiar Disease*, 2013; 11(11):1340-1341.	treatment group included fire-acupuncture
Song XF. Effectiveness of acupuncture in the treatment of patients with insomnia accompanying with depressive disorder [article in Chinese]. *China Health*	no diagnostic criteria for depression

Care & Nutrition, 2020; 30(13):330.	
Su L. Different acupuncture methods for the treatment of comorbid depression and sleep disorder in *Heart-Spleen* deficiency pattern: A randomized controlled trial [article in Chinese]. *Master thesis*, 2013; Xinjiang Medical University.	control group included acupuncture
Tan YF. Clinical study on the treatment of depression-induced insomnia by acupuncture combined with psychological counseling [article in Chinese]. *Master thesis*, 2012; Changchun University of Chinese Medicine.	treatment group included psychotherapy while control group did not include same therapy
Wang JJ, Liu ZF, Wang XC. Clinical study of acupuncture in the treatment of depression-related insomnia [article in Chinese]. *China's Naturopathy*, 2015(7):11-12.	no validated outcome indicators (sleep scales/questionnaires)
Wang J, Jiang JF, Wang LL. Clinical observation of "Governor-Vessel guiding *Qi*" method in the treatment of depression-related insomnia [article in Chinese]. *Chin Acup Moxib*, 2006; 26(5):328-330.	inconsistent use of Western medication in the control group patients resulted in an invalid comparison between acupuncture and Western medication
WLL, Zhang QL, Fan YJ, Lu XM, Yu H, Ren L. Clinical study of acupuncture with circular method on depression-related insomnia [article in Chinese]. *Xinjiang Journal of Traditional Chinese Medicine*, 2012; 30(4):42-44.	PSQI information was provided in only one group
Wang TJ, Wang LL, Tao WJ, Chen L. Clinical study on combined needle-embedding and medication for depressive sleep disorder [article in Chinese]. *Shanghai J Acup Moxib*, 2008; 27(5):5-7.	inconsistent use of Western medication in the control group patients resulted in an invalid comparison between acupuncture and Western medication
Wang TJ, Wang LL, Tao WJ, Chen L. Clinical study on combined needle-embedding and medication for depressive sleep disorder. *J Acupunct Tuina Sci*, 2009; 7(4):210-212.	inconsistent use of Western medication in the control group patients resulted in an invalid comparison between acupuncture and Western medication
Wang XH. Clinical efficacy of acupuncture with "Regulating *Qi* and Relieving Stagnation" method in the treatment of depression-related insomnia in the fire derived from stagnation of *Liver-Qi* pattern [article in Chinese]. *Master thesis*, 2020; Liaoning University of Traditional Chinese Medicine.	treatment- and control- groups included Chinese herbal medicine
Wang XJ, Wang LL, Qiao HF, Li JB. Clinical efficacy of combined acupuncture and medication in the treatment of depression-related sleep disorders [article in Chinese]. *Journal of Clinical Acupuncture and Moxibustion*, 2008; 24(12):1-2.	inconsistent use of Western medication in the control group patients resulted in an invalid comparison between acupuncture and Western medication
Wang YY, Fan R. The efficacy of combining acupuncture and medicine in treating 32 cases of comorbid depression and insomnia [article in Chinese]. *Journal of New Chinese Medicine*, 2011; 43(11):95-96.	non-RCT
Wang YM. Application and efficacy analysis of acupuncture in the treatment of depression-related insomnia [article in Chinese]. *Women's Health Research*,	inconsistent use of Western medication in the control group

2019; 22: 95-97.	patients resulted in an invalid comparison between acupuncture and Western medication
Wang Y. Clinical study of electroacupuncture for depression-related insomnia [article in Chinese]. *Master thesis*, 2018; Guangzhou University of Chinese Medicine.	control group included acupuncture
Wen X, Wu Q, Liu J, Xu Z, Fan L, Chen X, He Q, Ma R, Wu Y, Jiang S, Xu S, Fu W. Randomized single-blind multicenter trial comparing the effects of standard and augmented acupuncture protocols on sleep quality and depressive symptoms in patients with depression. *Psychol Health Med*, 2018; 23(4):375-390.	control group included acupuncture
Wen XY. Clinical study on the treatment of depression-related insomnia by acupuncture at eight confluence point [article in Chinese]. *Master thesis*, 2011; Guangzhou University of Chinese Medicine.	control group included acupuncture
Wu Q. A real-world study of acupuncture in improving sleep quality in patients with mild to moderate depression [article in Chinese]. *Doctorate thesis*, 2016; Guangzhou University of Chinese Medicine.	non-RCT
Wu XY. Clinical study on the treatment of depression-related insomnia by blood-letting theapy on Taiyang acupoint combined with Paroxetine [article in Chinese]. *Master thesis*, 2021; Anhui University of Chinese Medicine.	treatment group included "Three-edged needle" and cupping therapy
Wu YN. Clinical study on the treatment of depression-related insomnia with acupuncture with "Soothing *Liver* and Regulating Spirit" method [article in Chinese]. *Doctorate thesis*, 2016; Guangzhou University of Chinese Medicine.	treatment group included intradermal needle
Xu DH, Han RH, Li Z, Liu Q. Clinical efficacy of Fluoxetine combined with acupuncture in the treatment of patients with depression-related insomnia [article in Chinese]. *Proceedings of the 11th Annual Academic Conference of the Specialist Committee on Mental Illness of Chinese Society of Integrative Medicine*, 2012: 240-242.	non-RCT
Yan X, Zheng P, Zhang YN, Gao WN, Wen Q. Effect of acupuncture combined with repeated transcranial magnetic stimulation on sleep quality and depression in patients with primary insomnia comorbid with depression [article in Chinese]. *China Health Care & Nutrition*, 2020; 30(33):329-330.	patients could not be diagnosed as depression based on pre-treatment Self-Rating Depression Scale (SDS) scores
Yang CB, Wu JS, Li YT. The efficacy of electroacupuncture combined with Fluoxetine in treating 34 cases of insomnia caused by depression [article in Chinese]. *Jilin J Tradit Chin Med*, 2007; 27(12):42.	control group included acupuncture
Yao SP, Ma DX. Clinical study of acupuncture combined with medication in the treatment of depression with sleep disorder [article in Chinese]. *China Health Care & Nutrition*, 2016; 26(19)60-62.	treatment group included moxibustion
Ye JS. Clinical study on the treatment of depression-related insomnia by abdominal acupuncture [article in Chinese]. *Master thesis*, 2009; Guangzhou University of Chinese Medicine.	control group included acupuncture
You B. Clinical observation and systematic review of the effect of acupuncture in treating patients with insomnia accompanying with depressive disorder	no diagnostic criteria for depression

[article in Chinese]. *Chinese and Foreign Medical Research*, 2017; 15(30):44-45.	
Zhang J, Liu Y. Clinical effects of acupuncture with psychological counseling in the treatment of depression-induced insomnia [article in Chinese]. *Home Medicine*, 2019; 3:96-97.	treatment group included psychotherapy while control group did not include same therapy
Zhang J, Shen XZ. Effect of acupuncture on sleep quality and withdrawal symptoms due to discontinuation of medication in patients with mild to moderate depression [article in Chinese]. *International Journal of Traditional Chinese Medicine*, 2019; 7:706-710.	non-RCT
Zhang LX, Hui RT, Tang Y, Shi YZ, Xiao XJ, Zhou SY, Zheng QH, Cao W, Liu Y, Deng YL, Yu SY, Hu YP, Li Y. Effect of acupuncture intervention on insomnia with depression [article in Chinese]. *China Journal of Traditional Chinese Medicine and Pharmacy*, 2020; 35(8):4271-4274.	no diagnostic criteria for depression
Zhang R, Liu Y, Jia W, Shen J. 55 cases of depression-related insomnia treated by acupuncture [article in Chinese]. *Proceedings of the 10th Annual Academic Conference of the Specialist Committee on Mental Illness of Chinese Society of Integrative Medicine*, 2010: 43-45.	non-RCT
Zhang WL. A study on the relationship between *de-qi* and the efficacy in acupuncture with "Soothing *Liver* and Regulating Spirit" method on depression-induced insomnia [article in Chinese]. *Master thesis*, 2018; Guangzhou University of Chinese Medicine.	treatment groups included intradermal needle therapy
Zhang WL, Wang XF, Xuan J. 30 cases of depression-induced insomnia treated by acupuncture combined with psychological counseling [article in Chinese]. *Chinese Medicine Modern Distance Education of China*, 2017; 15(19):117-119.	treatment group included psychotherapy while control group did not include same therapy
Zhang YL. A study on the efficacy of acupuncture combined with agomelatine in depression accompanying with insomnia [article in Chinese]. *Proceedings of the 12th Annual National Academic Conference of the China Sleep Research Society*, 2020:2.	only abstract is available (no access to full article with data)
Zhen J, Yao XL, Chen T, Yang YJ, Zhou SH, Geng JH, Chen ZQ. Acupuncture combined with ear point embedding for sleep disorder in depression. *World Journal of Acupuncture-Moxibustion*, 2011; 21(3):30-34.	treatment group included auricular therapy
Zheng J, Zhu YH, Jiang RS, Jin HM. Clinical observation of electroacupuncture combined with transcranial direct current stimulation in treatment of senile depression-related insomnia [article in Chinese]. *Journal of Gannan Medical University*, 2017; 37(2):246-248.	no diagnostic criteria for depression
Zhong YS. Treatment of comorbid depression and insomnia by the acupoint prescription with "Tonifying *Kidney* and Regulating Spirit" method [article in Chinese]. *Master thesis*, 2011; Guangzhou University of Chinese Medicine.	treatment group included moxibustion
Zhou QB, Huang JY, Zhao J, Chen X, Zhang H. The effect of acupuncture treatment with the "Ziwu-Liuzhu-Najia" method on depression and sleep efficiency in patients with perimenopausal insomnia [article in Chinese]. *Hunan J Tradit Chin Med*, 2019; 35(05):77-79.	control group included acupuncture and Chinese herbal medicine

Appendix 3 Incidence of adverse events associated with each intervention

Adverse events	Involved RCTs	Incidence in different interventions			
		acupuncture	placebo- or sham-acupuncture	hypnotics and/or antidepressants	acupuncture + hypnotics and/or antidepressants
hand numbness and/or pain at acupoints	Chung et al. [26], Yin et al. [27], Yeung et al. [37]	22/116 (18.97%)	11/142 (7.75%)	Ø	Ø
fatigue	Chung et al. [26], Wang and Liu [47], Ye and Yan [49], Sun et al. [52]	8/60 (13.33%)	4/90 (4.44%)	3/85 (3.53%)	1/20 (5%)
headache	Chung et al. [26], Yeung et al. [37]	10/86 (11.63%)	12/142 (8.45%)	Ø	Ø
hematoma	Yin et al. [27], Yeung et al. [37], Chen [41]	3/34 (8.82%)	2/112 (1.79%)	0	Ø
fainting	Lin and Wang [43], Liu [46]	4/60 (6.67%)	Ø	0	Ø
gastro-intestinal symptoms (e.g., nausea, increased appetite, loss of appetite/poor appetite, diarrhea, constipation, etc.)	Chung et al. [26], Chen [41], Lin and Wang [43], Liu [46], Ye and Yan [49], Sun et al. [52], Min and Zhu [55]	3/60 (5%)	2/90 (2.22%)	38/183 (20.77%)	4/50 (8%)
dizziness	Chung et al. [26], Yin et al. [27], Yeung et al. [37], Liu [46], Wang and Liu [47], Ye and Yan [49], Min and Zhu [55]	4/86 (4.65%)	5/202 (2.48%)	7/145 (4.83%)	Ø

worsening of insomnia	Yeung et al. [37]	0	1/52 (1.92%)	Ø	Ø
palpitation	Yeung et al. [37]	0	1/52 (1.92%)	Ø	Ø
abnormal blood or biochemical indicators (e.g., abnormal liver function, leukocytopenia, abnormal metabolism of blood fat)	Wang et al. [48]	0	Ø	5/37 (13.51%)	Ø
excessive daytime sleepiness	Wang and Liu [47], Ye and Yan [49], Sun et al. [52], Min and Zhu [55]	0	Ø	8/105 (7.62%)	2/50 (4%)
sweating	Min and Zhu [55]	Ø	Ø	2/30 (6.67%)	1/30 (3.33%)
dry mouth	Liu [46], Wang and Liu [47], Ye and Yan [49], Min and Zhu [55]	0	Ø	6/115 (5.22%)	1/30 (3.33%)
elevated blood pressure	Sun et al. [52]	0	Ø	1/20 (5%)	2/20 (10%)
weight gain	Ye and Yan [49]	0	Ø	1/40 (2.5%)	Ø

***Notes*: "Ø" for where the adverse events were not measured**

Appendix 4 Methodological quality assessment of 21 included RCTs

Author, year	Random sequence generation	Allocation concealment	Blinding of participants	Blinding of personnel	Blinding of outcome assessment	Incomplete outcome data	Selective outcome reporting	Other bias (baseline balance)	Other bias (funding or conflict of interest)	Modified Jadad Score
Yin et al. [27]	L	L	L	H	L	L	L	L	L	5
Qin et al. [38]	L	U	L	H	U	L	U	L	U	4
Zhao et al. [39]	L	L	L	H	U	L	U	L	L	4
Chen et al. [40]	L	L	H	H	U	L	U	L	L	3
Chen [41]	U	U	H	H	U	L	U	L	L	2
He [42]	L	U	H	H	U	L	U	L	U	3
Lin and Wang [43]	L	U	H	H	U	L	U	L	L	3
Lin [44]	L	L	H	H	U	H	U	L	L	2
Liu [45]	L	U	H	H	U	L	U	L	L	3
Liu [46]	L	L	H	H	U	L	U	L	U	3
Wang and Liu [47]	U	U	H	H	U	L	U	L	L	2
Wang et al. [48]	L	U	H	H	L	L	U	L	L	4
Ye and Yan [49]	U	U	H	H	U	L	U	L	U	2
Liu and Li [50]	U	U	H	H	U	L	U	L	U	2
Liu et al. [51]	L	U	H	H	U	L	U	L	U	3
Sun et al. [52]	L	U	H	H	U	L	U	L	U	3
Tan et al. [53]	L	U	H	H	U	L	U	L	U	3
Wang and Ai [54]	L	U	H	H	U	L	U	L	U	3
Min and Zhu [55]	L	U	H	H	U	L	U	L	U	3
Chung et al. [26]	L	L	L	H	L	L	L	L	L	5
Yeung et al. [37]	L	L	L	H	L	L	L	L	L	5

Abbreviations: L, Low risk; U, Unclear risk; H, High risk

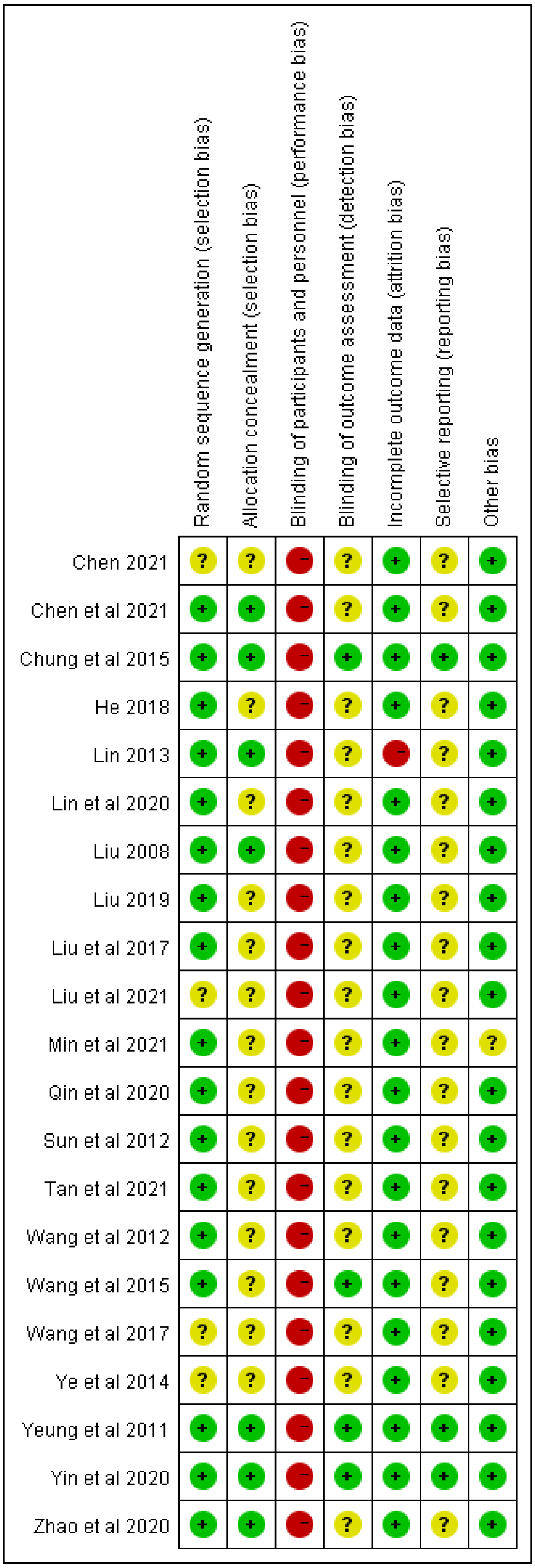

Appendix 5 Risk of bias summary

Appendix 6 Details of acupuncture procedure based on revised STRICTA (2010 Version)

Item of STRICTA	*1. Acupuncture rationale*			*2. Needling details*							*3. Treatment regimen*		*4. Other components of treatment*		*5. Practitioner background*	6. Control or comparator interventions	
	(1a) Style of acupuncture	(1b) Rationale for treatment	(1c) Extent to which treatment was varied	(2a) Number of needles inserted	(2b) points used	(2c) Depths of insertion	(2d) Responses elicited	(2e) Needle stimulation	(2f) Needle retention time	(2g) Needle type	(3a) Number of treatment sessions	(3b) Frequency and duration of treatment sessions	(4a) Details of other interventions administered to the acupuncture group	(4b) Setting and context of treatment	Description of acupuncturist	(6a) Rationale for the control or comparator	(6b) Precise description of the control or comparator
Studies eligible to the items	Chung et al. [26], Yin et al. [27], Yeung et al. [37], Qin et al. [38], Zhao et al. [39], Chen et al. [40], Chen [41], He [42], Lin and Wang [43], Lin [44], Liu [45], Liu	Chung et al. [26], Yin et al. [27], Yeung et al. [37], Qin et al. [38], Zhao et al. [39], Chen et al. [40], Chen [41], He [42], Lin and Wang [43], Lin [44], Liu [45], Liu	N/A	Chung et al. [26], Yin et al. [27], Yeung et al. [37], Qin et al. [38], Zhao et al. [39], Chen et al. [40], Chen [41], He [42], Lin and Wang [43], Lin [44], Liu [45], Liu	Chung et al. [26], Yin et al. [27], Yeung et al. [37], Qin et al. [38], Zhao et al. [39], Chen et al. [40], Chen [41], He [42], Lin and Wang [43], Lin [44], Liu [45], Liu [46], Wang and Liu [47], Wang	Chung et al. [26], Yin et al. [27], Zhao et al. [39], Chen [41], Lin and Wang [43], Lin [44], Liu [45],	Chung et al. [26], Yin et al. [27], Yeung et al. [37], Qin et al. [38], Zhao et al. [39], Chen et al. [40], Chen [41], He [42], Lin and Wang [43], Lin [44], Liu [45], Liu	Chung et al. [26], Yin et al. [27], Yeung et al. [37], Qin et al. [38], Zhao et al. [39], Chen et al. [40], Chen [41], He [42], Lin and Wang [43], Lin [44], Liu [45], Liu	Chung et al. [26], Yin et al. [27], Yeung et al. [37], Qin et al. [38], Zhao et al. [39], Chen et al. [40], Chen [41], He [42], Lin and Wang [43], Lin [44], Liu [45], Liu	Chung et al. [26], Yin et al. [27], Yeung et al. [37], Qin et al. [38], Zhao et al. [39], Chen et al. [40], Chen [41], He [42], Lin [44], Liu	Chung et al. [26], Yin et al. [27], Yeung et al. [37], Qin et al. [38], Zhao et al. [39], Chen et al. [40], Chen [41], He [42], Lin and Wang [43], Lin [44], Liu [45], Liu [46], Wang and Liu [47], Wang	Chung et al. [26], Yin et al. [27], Yeung et al. [37], Qin et al. [38], Zhao et al. [39], Chen et al. [40], Chen [41], He [42], Lin and Wang [43], Lin [44], Liu [45], Liu [46], Wang and Liu [47], Wang	Chung et al. [26], Yin et al. [27], Yeung et al. [37], Qin et al. [38], Liu and Li [50], Liu et	Chung et al. [26], Yin et al. [27], Yeung et al. [37]	Chung et al. [26], Yin et al. [27], Yeung et al. [37]	Chung et al. [26], Yin et al. [27], Yeung et al. [37], Qin et al. [38], Zhao et al. [39], Chen et al. [40], Chen [41], He [42], Lin and Wang [43], Lin [44], Liu [45], Liu [46], Wang and Liu [47], Wang	Chung et al. [26], Yin et al. [27], Yeung et al. [37], Qin et al. [38], Zhao et al. [39], Chen et al. [40], Chen [41], He [42], Lin and Wang [43], Lin [44], Liu [45], Liu [46], Wang and Liu [47], Wang

		[46], Wang and Liu [47], Wang et al. [48], Ye and Yan [49], Liu and Li [50], Liu et al. [51], Sun et al. [52], Tan et al. [53], Wang and Ai [54], Min and Zhu [55]	[46], Wang and Liu [47], Wang et al. [48], Ye and Yan [49], Liu and Li [50], Liu et al. [51], Sun et al. [52], Tan et al. [53], Wang and Ai [54], Min and Zhu [55]		[46], Wang and Liu [47], Wang et al. [48], Ye and Yan [49], Liu and Li [50], Liu et al. [51], Sun et al. [52], Tan et al. [53], Wang and Ai [54], Min and Zhu [55]	et al. [48], Ye and Yan [49], Liu and Li [50], Liu et al. [51], Sun et al. [52], Tan et al. [53], Wang and Ai [54], Min and Zhu [55]	Liu [46], Wang et al. [48]	[46], Wang and Liu [47], Wang et al. [48], Ye and Yan [49], Liu and Li [50], Liu et al. [51], Sun et al. [52], Tan et al. [53], Wang and Ai [54], Min and Zhu [55]	[46], Wang and Liu [47], Wang et al. [48], Ye and Yan [49], Liu and Li [50], Liu et al. [51], Sun et al. [52], Tan et al. [53], Wang and Ai [54], Min and Zhu [55]	[46], Wang and Liu [47], Wang et al. [48], Ye and Yan [49], Liu et al. [51], Sun et al. [52], Tan et al. [53], Wang and Ai [54]	[46], Wang and Liu [47], Wang et al. [48], Ye and Yan [49], Liu et al. [51], Tan et al. [53], Wang and Ai [54]	et al. [48], Ye and Yan [49], Liu and Li [50], Liu et al. [51], Sun et al. [52], Tan et al. [53], Wang and Ai [54], Min and Zhu [55]	et al. [48], Ye and Yan [49], Liu and Li [50], Liu et al. [51], Sun et al. [52], Tan et al. [53], Wang and Ai [54], Min and Zhu [55]	al. [51], Sun et al. [52], Tan et al. [53], Wang and Ai [54], Min and Zhu [55]			et al. [48], Ye and Yan [49], Liu and Li [50], Liu et al. [51], Sun et al. [52], Tan et al. [53], Wang and Ai [54], Min and Zhu [55]	et al. [48], Ye and Yan [49], Liu and Li [50], Liu et al. [51], Sun et al. [52], Tan et al. [53], Wang and Ai [54], Min and Zhu [55]
Total number and percentage [n (%)]		21 (100)	21 (100)	0 (0)	21 (100)	21 (100)	9 (42.9)	21 (100)	21 (100)	19 (90.5)	16 (76.2)	21 (100)	21 (100)	10 (47.6)	3 (14.3)	3 (14.3)	21 (100)	21 (100)
Details of acupuncture procedure in each trial	Yin et al. [27]	Chinese Acup	TCM theory	NR	Reported	EX, EX-HN3, GV20, GV24, HT7, PC6, SP6	0.5-1 *cun*	*De-qi*	EA	30 min	stainless steel (0.30*25 and 0.30*40 mm)	8 weeks	30 min/session, 3 sessions/week (once every other day) for 8 weeks (continuous wave, 30Hz, 0.1-1mA)	routine antidepressant were allowed	Reported	> 5 years of experience	sham-EA and placebo-EA	Reported (details in Table 1)
	Qin et al. [38]	Chinese Acup	TCM theory	NR	Reported	BL18, EX, EX-HN1, EX-HN3, HT7, GV20, KI6, SP6	NR	*De-qi*	MA	30 min	stainless steel (0.30*25 and 0.30*40 mm)	4 weeks	30 min/session, 3 - 4 sessions/week (once every other day) for 4 weeks (Fluoxetine + Deanxit as basic treatment)	Fluoxetine + Deanxit as basic treatment	NR	NR	placebo-MA	Reported (details in Table 1)

	Zhao et al. [39]	Chinese Acup	TCM theory	NR	Reported	EX-HN1, GB13, GV11, GV24, HT7	10-30 mm	*De-qi*	MA	30 min	stainless steel (0.25*25 mm)	8 weeks	30 min/session, 3 sessions/week for 8 weeks	NR	NR	NR	placebo-MA	Reported (details in Table 1)
	Chen et al. [40]	Chinese Acup	TCM theory	NR	Reported	EX-HN1, GB20, Gongxue (1.5 *Cun* below GB20), GV20, cluster needling on frontal region	NR	*De-qi*	EA	25 min	stainless steel (0.30*40 mm)	6 weeks	25 min/session, 5 sessions/week for 6 weeks (sparse wave, 2Hz)	NR	NR	NR	Paroxetine	Reported (details in Table 1)
	Chen [41]	Chinese Acup	TCM theory	NR	Reported	EX-HN3, GV20, BL13, BL15, BL18, BL20, BL23	0.3-0.8 *cun*	*De-qi*	EA	30 min	stainless steel (0.25*40 mm)	4 weeks	30 min/session, 7 sessions/week for 4 weeks (continuous wave, 2Hz, 0.6mA)	NR	NR	NR	Sertraline	Reported (details in Table 1)
	He [42]	Chinese Acup	TCM theory	NR	Reported	GV20, HT7, PC6, Zhenjing, Shangen	NR	*De-qi*	MA	30 min	stainless steel (0.1*9.5 cm)	6 weeks	30 min/session, 7 sessions/week for 6 weeks	NR	NR	NR	Paroxetine	Reported (details in Table 1)
	Lin and Wang [43]	Chinese Acup	TCM theory	NR	Reported	EX-HN1, EX-HN3, GV20, HT7, LR3, PC6, PC7	0.5-1 *cun*	*De-qi*	MA	30 min	NR	4 weeks	30 min/session, 5 sessions/week for 4 weeks	NR	NR	NR	Escitalopram	Reported (details in Table 1)
	Lin [44]	Chinese Acup	TCM theory	NR	Reported	EX-HN1, EX-HN3, GV20, HT7, PC6, SP6	0.5-1 *cun*	*De-qi*	EA	20 min	stainless steel (0.30*25 mm and 0.25*40 mm)	24 weeks	20 min/session, 3 sessions/week for 6 weeks + 2 sessions/week for 6 weeks + 1 session/week for 12 weeks (intermittent wave, 40Hz)	NR	NR	NR	Citalopram	Reported (details in Table 1)

	Liu [45]	Chinese Acup	TCM theory	NR	Reported	CV6, CV10, CV12, CV13, EX-HN3, GV20, GV24, PC6, ST25, ST36	0.5-1.5 *cun*	De-qi	MA	30 min	NR	7.5 weeks	30 min/session, 4 sessions/week for 7.5 weeks	NR	NR	NR	Fluoxetine + Eszopiclone	Reported (details in Table 1)
	Liu [46]	Chinese Acup	TCM theory	NR	Reported	0.5 *Cun* next to EX-HN1, 0.5 *Cun* up to EX-HN3, 0.5 *Cun* up to GB14, BL62, KI6, PC6, SP6	0.5-1 *cun*	*De-qi*	MA	30 min	stainless steel (0.30*40 mm)	3 weeks	30 min/session, 6 sessions/week for 3 weeks	NR	NR	NR	Clonazepam	Reported (details in Table 1)
	Wang and Liu [47]	Chinese Acup	TCM theory	NR	Reported	EX-HN3, GV20, HT7, LR3, SP6, ST36	NR	*De-qi*	MA	30 min	stainless steel (0.30*40 mm)	12 weeks	30 min/session, 3 - 4 sessions/week (once every other day) for 12 weeks	NR	NR	NR	Mirtazapine	Reported (details in Table 1)
	Wang et al. [48]	Chinese Acup	TCM theory	NR	Reported	EX-HN3, GV20, HT7, LI4, LR3	5 mm-20 mm	*De-qi*	MA	30 min	stainless steel (0.30*25 mm)	4 weeks	30 min/session, 6 sessions/week for 4 weeks	NR	NR	NR	Mirtazapine	Reported (details in Table 1)
	Ye and Yan [49]	Chinese Acup	TCM theory	NR	Reported	EX-HN3, HT7, LR3, SP6, ST36	NR	*De-qi*	MA	30 min	stainless steel (0.30*40 mm)	12 weeks	30 min/session, 3-4 sessions/week (once every other day) for 12 weeks	NR	NR	NR	Mirtazapine	Reported (details in Table 1)
	Liu and Li [50]	Chinese Acup	TCM theory	NR	Reported	GV20, GV26, HT7, LR3, PC6, SP6	NR	*De-qi*	MA	NR	NR	12 weeks	5 sessions/week for 12 weeks	Venlafaxin e	NR	NR	Venlafaxine	Reported (details in Table 1)
	Liu et al. [51]	Chinese Acup	TCM theory	NR	Reported	BL62, EX-HN1, GV20, HT7, KI6, LR3, PC6	NR	*De-qi*	MA	30 min	stainless steel (0.30*40 mm)	4 weeks	30 min/session, 7 sessions/week for 4 weeks	Mirtazapin e	NR	NR	Mirtazapine	Reported (details in Table 1)
	Sun et	Chinese Acup	TCM theory	NR	Reported	EX-HN3, GV20, PC6,	NR	De-qi	EA	30 min	NR	2 weeks	30 min/session, 5	Venlafaxin	NR	NR	Venlafaxine	Reported (details in

	al. [52]					ST36							sessions/week for 2 weeks	e				Table 1)
	Tan et al. [53]	Chinese Acup	TCM theory	NR	Reported	EX-HN1, HT7, LR3, SP6	NR	*De-qi*	MA	30 min	stainless steel (0.30*25 mm)	6 weeks	30 min/session, 3-4 sessions/week (once every other day) for 6 weeks	Paroxetine	NR	NR	Paroxetine	Reported (details in Table 1)
	Wang and Ai [54]	Chinese Acup	TCM theory	NR	Reported	EX-HN3, GV20, HT7, PC6, SP6, ST36	NR	*De-qi*	EA	30 min	stainless steel (0.30*40 mm)	4 weeks	30 min/session, 7 sessions/week for 4 weeks (continuous wave)	Paroxetine	NR	NR	Paroxetine	Reported (details in Table 1)
	Min and Zhu [55]	Chinese Acup	TCM theory	NR	Reported	EX-HN3, GB20, GV14, GV16, GV20, PC6, SP6	NR	*De-qi*	MA	NR	NR	6 weeks	3 sessions/week for 6 weeks	Paroxetine	NR	NR	Paroxetine	Reported (details in Table 1)
	Chung et al. [26]	Chinese Acup	TCM theory	NR	Reported	EX, EX-HN1, EX-HN3, GV20, HT7, PC6, SP6, TF_4	2-25 mm	*De-qi*	EA	30 min	stainless steel (0.25*25 mm)	3 weeks	30 min/session, 3 sessions/week for 3 weeks (square wave, 4-Hz)	routine antidepres sant were allowed	Report ed	≥ 3 years of experie nce	sham-EA and placebo-EA	Reported (details in Table 1)
	Yeung et al. [37]	Chinese Acup	TCM theory	NR	Reported	EX, EX-HN1, EX-HN3, GV20, TF_4	NR	*De-qi*	EA	30 min	stainless steel (0.25*25 mm)	3 weeks	30 min/session, 3 sessions/week for 3 weeks (square wave, 4-Hz)	routine antidepres sant were allowed	Report ed	3 years of experie nce	sham-EA and placebo-EA	Reported (details in Table 1)

Notes: The *cun* is a traditional Chinese unit of length equal to the width of a patient's thumb at the knuckle; *De-qi* (obtaining Qi) refers to acupuncture-evoked specific sensations such as soreness, numbness, heaviness, and distention at the site of needle placement, and these sensations may spread to other parts of the body

Abbreviations: NR, no record; Acup, acupuncture; TCM, Traditional Chinese Medicine; MA, manual acupuncture; EA, electroacupuncture; BL13, Feishu; BL15, Xinshu; BL18, Ganshu; BL20, Pishu; BL23, Shenshu; BL62, Shenmai; CV6, Qihai, CV10, Xiawan; CV12,

Zhongwan; CV13, Shangwan; EX, Anmian; EX-HN1, Sishencong; EX-HN3, Yintang; GB13, Benshen; GB14, Yangbai; GB20, Fengchi; GV11, Shendao; GV14, Dazhui; GV16, Fengfu; GV20, Baihui; GV24, Shenting; GV26, Shuigou; HT7, Shenmen; KI6, Zhaohai; LI4, Hegu; LR3, Taichong; PC6, Neiguan; PC7, Daling; SP6, Sanyingjiao; ST25, Tianshu; ST36, Zusanli; TF4, Ear Shenmen

Appendix 7 Qualitative and quantitative analyses in the 21 included studies

Types of insomnia	Interventions *Vs.* Controls		Number of studies	Qualitative analysis	Quantitative analysis				Publication bias (n ≥ 10)
					pooled effect sizes (n ≥ 3)	subgroup analysis (n ≥ 3)	sensitivity analysis (n ≥ 10)	meta-regression analysis (n ≥ 10)	
major	Acup *Vs.* placebo-/sham- Acup	Acup *Vs.* placebo-Acup	3	All	(i) PSQI: 3 studies<br>(ii) HAMD: 3 studies	Ø	Ø	Ø	(i) PSQI: 20 studies<br>(ii) HAMD: 18 studies
		Acup *Vs.* sham-Acup	1	All	Ø	Ø	Ø	Ø	
	Acup *Vs.* antidepressant and/or hypnotic		10	All	(i) PSQI: 10 studies<br>(ii) HAMD: 7 studies<br>(iii) SDS: 3 studies<br>(iv) six domains of PSQI: 3 studies<br>(v) total clinical effectiveness rate: 9 studies	(i) PSQI<br>(ii) HAMD	PSQI	PSQI	
	Acupuncture + (antidepressant and/or hypnotic) *Vs.* (antidepressant and/or hypnotic)		6	All	(i) PSQI: 5 studies<br>(ii) HAMD: 6 studies<br>(iii) total clinical effectiveness rate: 5 studies	(i) PSQI<br>(ii) HAMD<br>(iii) total clinical effectiveness	Ø	Ø	
residual	Acup *Vs.* placebo-/sham- Acup	Acup *Vs.* placebo-Acup	2	All	Ø	Ø	Ø	Ø	
		Acup *Vs.* sham-Acup	2	All	Ø	Ø	Ø	Ø	

Notes: Type of insomnia (major = insomnia as a major symptom of depression; residual = insomnia as a residual symptom of depression); Ø, N/A

Abbreviations: Acup, acupuncture; PSQI, Pittsburgh Sleep Quality Index; HAMD, Hamilton Depression Scale; SDS, Self-Rating Depression Scale

Appendix 8 Criteria of total clinical effectiveness rate reported in the included studies

Criteria	Details of guidelines	Involved studies	Frequency, n (%)
**Method 1**	***Healed***: reduction rate of PSQI and/or HAMD global scores ≥ 75%	Chen et al. [40], Chen [41], Lin and Wang [43], Liu [45], Wang and Liu [47], Wang et al. [48], Sun et al. [52], Tan et al. [53], Min and Zhu [55]	9 (60)
	Significant efficacious: 75% > reduction rate of PSQI and/or HAMD global scores ≥ 50%		
	Efficacious: 50% > reduction rate of PSQI and/or HAMD global scores ≥ 25%		
	Inefficacious: reduction rate of PSQI and/or HAMD global scores < 25%		
**Method 2**	***Healed***: at the end of treatment, sleep pattern is back to normal and more than 6 hours per night	He [42], Liu [46], Liu et al. [51], Wang and Ai [54]	4 (26.67)
	Significant efficacious: sleep pattern is clearly improved and sleep time is increased by more than 3 hours		
	Efficacious: symptoms are relieved and sleep time is increased by less than 3 hours		
	Inefficacious: symptoms were not relieved		
**Method 3**	***Healed***: all symptoms disappeared; reduction rate of PSQI global scores ≥ 85%	Qin et al. [38], Ye and Yan [49]	2 (13.33)
	Significant efficacious: symptoms alleviated; 85% > reduction rate of PSQI global scores ≥ 60%		
	Efficacious: symptoms slightly alleviated; 60% > reduction rate of PSQI global scores ≥ 30%		
	Inefficacious: symptoms were not relieved; reduction rate of PSQI global scores < 30%		

Notes: reduction rate of PSQI global scores = [(PSQI global scores at pre-treatment - PSQI global scores at post-treatment)/(PSQI global scores at pre-treatment)] X 100%; reduction rate of HAMD global scores = [(HAMD global scores at pre-treatment - HAMD global scores at post-treatment)/(HAMD global scores at pre-treatment)] X 100%

Abbreviations: PSQI, Pittsburgh Sleep Quality Index; HAMD, Hamilton Depression Scale

Appendix 9 Subgroup analyses of PSQI and HAMD (Acupuncture *Vs*. Western medication)

Basis for subgroup classification	All trials or subgroup title	No. of Studies	No. of participants	Statistical method	Effect size	*p*	I^2	Subgroup analysis results
PSQI	All trials	10	668	Mean Difference (IV, Random, 95% CI)	-1.17 [-2.26, -0.08]	0.03	91	
Acupuncture method	(i) MA	7	483	Mean Difference (IV, Random, 95% CI)	-1.12 [-2.40, 0.16]	0.09	93	Chi² statistic 0.06, df = 1, *p* = 0.81
	(ii) EA	3	185	Mean Difference (IV, Random, 95% CI)	-1.40 [-3.24, 0.45]	0.14	66	
Principle of acupuncture prescription	fixed	8	518	Mean Difference (IV, Random, 95% CI)	-1.16 [-2.49, 0.16]	0.09	91	Chi² statistic 0.00, df = 1, *p* = 0.97
	semi-standardised	2	150	Mean Difference (IV, Random, 95% CI)	-1.23 [-4.09, 1.63]	0.40	94	
Frequency of treatment	≥ 5 sessions per week	6	390	Mean Difference (IV, Random, 95% CI)	-1.76 [-3.40, -0.13]	0.03	93	Chi² statistic 4.15, df = 1, *p* = 0.04
	< 5 sessions per week	4	278	Mean Difference (IV, Random, 95% CI)	0.07 [-0.59, 0.73]	0.84	37	
needle retention time	≥ 30 minutes	8	550	Mean Difference (IV, Random, 95% CI)	-1.12 [-2.31, 0.07]	0.07	92	Chi² statistic 0.01, df = 1, *p* = 0.90
	< 30 minutes	2	118	Mean Difference (IV, Random, 95% CI)	-1.33 [-4.45, 1.80]	0.41	80	
Type of standard care	(i) *Vs*. antidepressant	7	478	Mean Difference (IV, Random, 95% CI)	-0.34 [-1.19, 0.50]	0.43	80	Chi² statistic 33.66, df = 2, *p* < 0.01
	(ii) *Vs*. hypnotic	1	60	Mean Difference (IV, Random, 95% CI)	-4.30 [-5.37, -3.23]	< 0.01	Ø	

	(iii) *Vs*. antidepressant + hypnotic	2	130	Mean Difference (IV, Random, 95% CI)	-2.59 [-3.59, -1.59]	< 0.01	0	
HAMD	All trials	7	496	Std. Mean Difference (IV, Random, 95% CI)	-0.47 [-0.91, -0.02]	0.04	83	
Acupuncture method	(i) MA	5	359	Std. Mean Difference (IV, Random, 95% CI)	-0.63 [-1.24, -0.02]	0.04	87	Chi² statistic 2.40, df = 1, *p* = 0.12
	(ii) EA	2	137	Std. Mean Difference (IV, random, 95% CI)	-0.08 [-0.42, 0.25]	0.63	0	
Principle of acupuncture prescription	fixed	5	346	Std. Mean Difference (IV, Random, 95% CI)	-0.64 [-1.24, -0.03]	0.04	87	Chi² statistic 2.63, df = 1, *p* = 0.10
	semi-standardised	2	150	Std. Mean Difference (IV, random, 95% CI)	-0.06 [-0.40, 0.28]	0.72	11	
Frequency of treatment	≥ 5 sessions per week	4	266	Std. Mean Difference (IV, Random, 95% CI)	-0.65 [-1.46, 0.16]	0.11	90	Chi² statistic 0.82, df = 1, *p* = 0.36
	< 5 sessions per week	3	230	Std. Mean Difference (IV, random, 95% CI)	-0.24 [-0.60, 0.12]	0.19	47	
needle retention time	≥ 30 minutes	6	426	Std. Mean Difference (IV, Random, 95% CI)	-0.51 [-1.04, 0.02]	0.06	86	Chi² statistic 0.52, df = 1, *p* = 0.47
	< 30 minutes	1	70	Std. Mean Difference (IV, random, 95% CI)	-0.25 [-0.72, 0.22]	0.30	Ø	
Type of standard care	(i) *Vs*. antidepressant	5	366	Std. Mean Difference (IV, Random, 95% CI)	-0.51 [-1.15, 0.13]	0.12	89	Chi² statistic 0.13, df = 1, *p* = 0.71
	(ii) *Vs*. antidepressant + hypnotic	2	130	Std. Mean Difference (IV, random, 95% CI)	-0.38 [-0.72, -0.03]	0.03	0	
Version of HAMD	(i) 17-item HAMD	6	424	Std. Mean Difference (IV, Random, 95% CI)	-0.48 [-1.01, 0.06]	0.08	86	Chi² statistic 0.01, df = 1, *p* = 0.92

	(ii) 24-item HAMD	1	72	Std. Mean Difference (IV, random, 95% CI)	-0.44 [-0.91, 0.03]	0.06	Ø	

Abbreviations: MA, manual acupuncture; EA, electroacupuncture; Ø, N/A

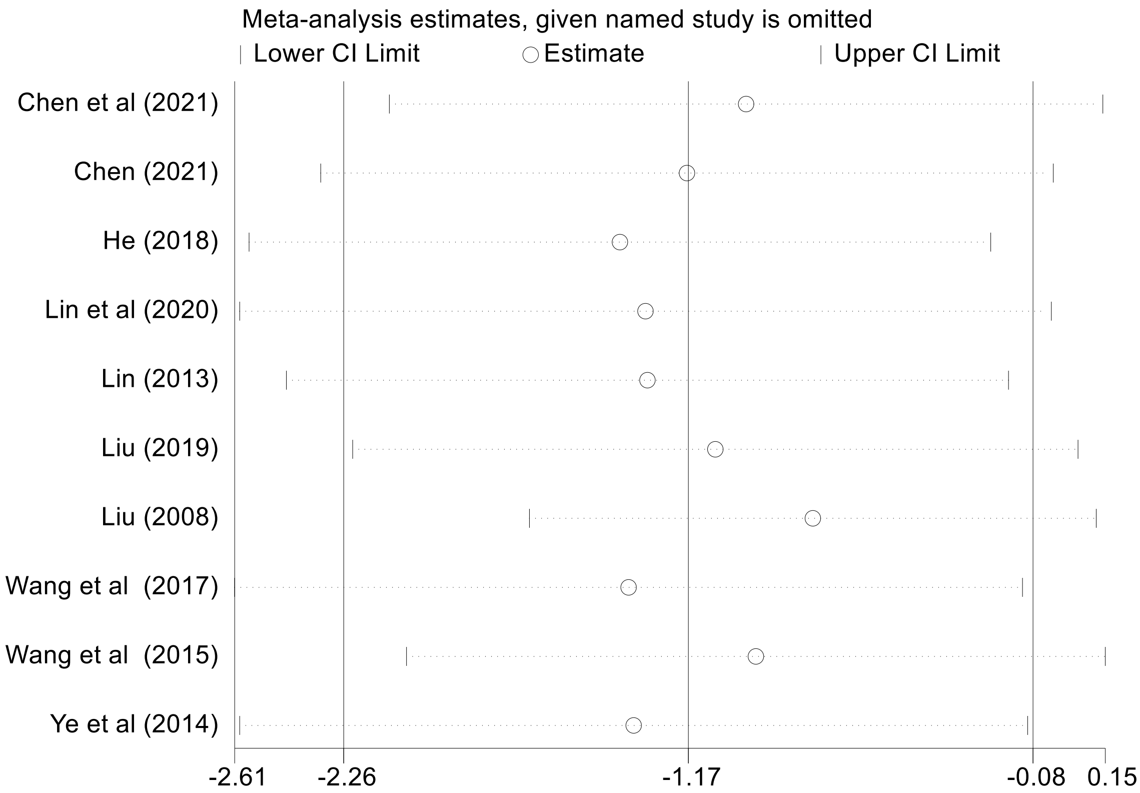

Appendix 10 Sensitivity analysis of PSQI global scores (Acupuncture *Vs.* Western medication)

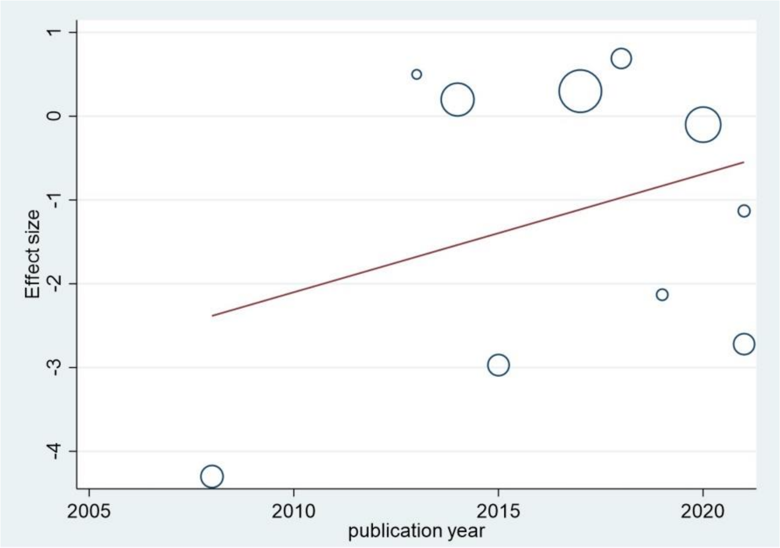

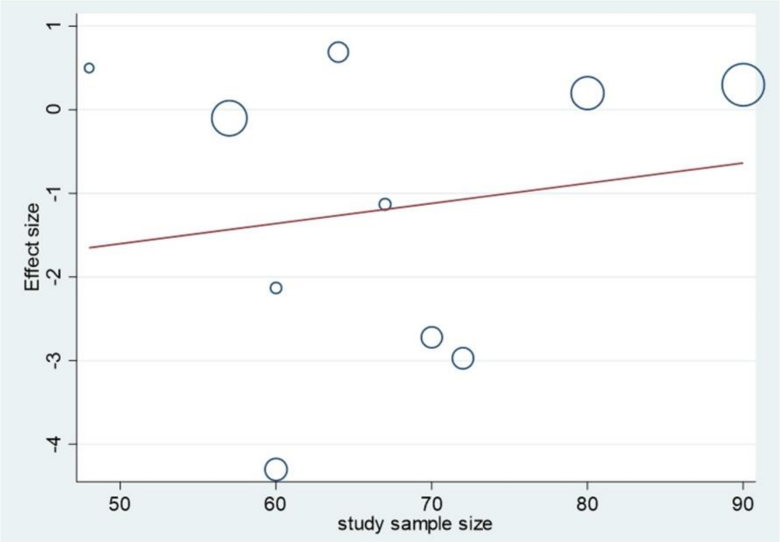

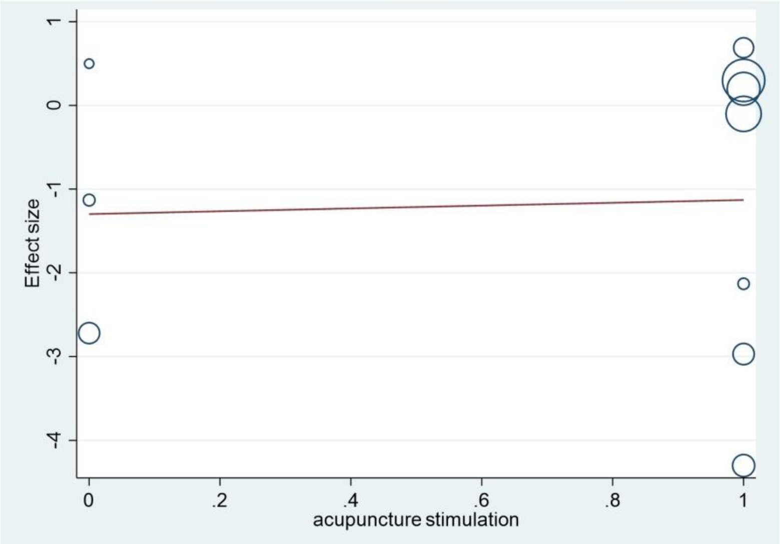

Appendix 11 Meta-regression analysis of PSQI global scores (Acupuncture *Vs.* Western medication)

Appendix 12 Subgroup analyses of PSQI, HAMD, total clinical effectiveness rate (Acupuncture + Western medication *Vs*. Western medication)

Basis for subgroup classification	All trials or subgroup title	No. of Studies	No. of participants	Statistical method	Effect size	*p*	I^2	Subgroup analysis results
PSQI	All trials	5	358	Mean Difference (IV, Random, 95% CI)	-2.99 [-4.22, -1.76]	< 0.01	91	
Acupuncture method	(i) MA	4	278	Mean Difference (IV, Random, 95% CI)	-3.03 [-4.64, -1.43]	< 0.01	93	Chi² statistic 0.03, df = 1, *p* = 0.87
	(ii) EA	1	80	Mean Difference (IV, Random, 95% CI)	-2.88 [-3.66, -2.10]	< 0.01	Ø	
Principle of acupuncture prescription	fixed	2	157	Mean Difference (IV, Random, 95% CI)	-2.04 [-2.59, -1.50]	< 0.01	0	Chi² statistic 2.13, df = 1, *p* = 0.14
	semi-standardised	3	201	Mean Difference (IV, Random, 95% CI)	-3.67 [-5.78, -1.56]	< 0.01	94	
Frequency of treatment	≥ 5 sessions per week	3	201	Mean Difference (IV, Random, 95% CI)	-2.30 [-2.87, -1.73]	< 0.01	38	Chi² statistic 0.82, df = 1, *p* = 0.37
	< 5 sessions per week	2	157	Mean Difference (IV, Random, 95% CI)	-4.12 [-8.03, -0.21]	0.04	97	
HAMD	All trials	6	398	Std. Mean Difference (IV, random, 95% CI)	-0.80 [-1.17, -0.44]	< 0.01	66	
Acupuncture method	(i) MA	4	278	Std. Mean Difference (IV, random, 95% CI)	-0.92 [-1.40, -0.43]	< 0.01	73	Chi² statistic 0.80, df = 1, *p* = 0.37
	(ii) EA	2	120	Std. Mean Difference (IV, random, 95% CI)	-0.59 [-1.05, -0.12]	0.01	33	
Principle of acupuncture prescription	fixed	3	197	Std. Mean Difference (IV, random, 95% CI)	-0.87 [-1.65, -0.09]	0.03	85	Chi² statistic 0.13, df = 1, *p* = 0.72

	semi-standardised	3	201	Std. Mean Difference (IV, random, 95% CI)	-0.72 [-1.01, -0.43]	< 0.01	0	
Frequency of treatment	≥ 5 sessions per week	4	241	Std. Mean Difference (IV, random, 95% CI)	-0.61 [-0.87, -0.35]	< 0.01	0	Chi² statistic 1.95, df = 1, *p* = 0.16
	< 5 sessions per week	2	157	Std. Mean Difference (IV, random, 95% CI)	-1.20 [-1.99, -0.42]	< 0.01	80	
Total clinical effectiveness rate	All trials	5	337	Risk Ratio (M-H, random, 95% CI)	1.11 [0.93, 1.33]	0.24	76	
Acupuncture method	(i) MA	3	217	Risk Ratio (M-H, random, 95% CI)	1.23 [1.05, 1.43]	< 0.01	28	Chi² statistic 4.69, df = 1, *p* = 0.03
	(ii) EA	2	120	Risk Ratio (M-H, random, 95% CI)	0.94 [0.77, 1.13]	0.51	52	
Principle of acupuncture prescription	fixed	3	197	Risk Ratio (M-H, random, 95% CI)	1.15 [1.04, 1.27]	< 0.01	0	Chi² statistic 0.07, df = 1, *p* = 0.80
	semi-standardised	2	140	Risk Ratio (M-H, random, 95% CI)	1.07 [0.64, 1.79]	0.79	89	
Frequency of treatment	≥ 5 sessions per week	3	180	Risk Ratio (M-H, random, 95% CI)	1.05 [0.80, 1.39]	0.71	79	Chi² statistic 0.57, df = 1, *p* = 0.45
	< 5 sessions per week	2	157	Risk Ratio (M-H, random, 95% CI)	1.20 [1.00, 1.43]	0.05	35	

Appendix 13 Evidence quality assessment of major outcome measures based on GRADE system

Interventions Vs. controls	Outcomes	Numbers of studies (numbers of participants)	Estimated effects (MD/SMD/RR with 95% CI)	I^2% (p)	Limitations	Inconsistency	Indirectness	Imprecision	Publication bias	Quality of evidence
acupuncture *Vs.* placebo-acupuncture	PSQI global scores	3 (277)	MD = -3.12, 95%CI (-5.16, -1.08)	84 (p < 0.01)	0	-1②	0	-1③	0	Low
	HAMD global scores	3 (277)	SMD = -2.67, 95%CI (-3.51, -1.84)	82 (p < 0.01)	0	-1②	0	-1③	0	Low
acupuncture *Vs.* psychotropic medication	PSQI global scores	10 (668)	MD= -1.17, 95%CI (-2.26, -0.08)	91 (p = 0.03)	-1①	-1②	0	0	0	Low
	subjective sleep quality scores	3 (179)	MD= -0.01, 95%CI (-0.23, 0.21)	0 (p = 0.94)	-1①	0	0	-1③	0	Low
	sleep latency scores	3 (179)	MD= 0.05, 95%CI (-0.15, 0.25)	0 (p = 0.64)	-1①	0	0	-1③	0	Low
	habitual sleep efficiency scores	3 (179)	MD= -0.14, 95%CI (-0.42, 0.13)	0 (p = 0.31)	-1①	0	0	-1③	0	Low
	sleep duration scores	3 (179)	MD= 0.01, 95%CI (-0.24, 0.25)	0 (p = 0.95)	-1①	0	0	-1③	0	Low
	sleep disturbances scores	3 (179)	MD= 0.03, 95%CI (-0.16, 0.22)	0 (p = 0.72)	-1①	0	0	-1③	0	Low
	daytime dysfunction scores	3 (179)	MD= 0.13, 95%CI (-0.11, 0.38)	35 (p = 0.29)	-1①	0	0	-1③	0	Low
	HAMD global scores	7 (496)	SMD= -0.47, 95%CI (-0.91, -0.02)	83 (p = 0.04)	-1①	-1②	0	0	0	Low
	SDS global scores	3 (172)	MD= 2.10, 95%CI (-4.20, 8.39)	88 (p = 0.51)	-1①	-1②	0	-1③	0	Very low
	total clinical effectiveness rate	9 (620)	RR = 1.09, 95%CI (1.02, 1.17)	26 (p = 0.01)	-1①	0	0	0	0	Moderate
acupuncture + psychotropic medication Vs. psychotropic medication	PSQI global scores	5 (358)	MD= -2.99, 95%CI (-4.22, -1.76)	91 (p < 0.01)	-1①	-1②	0	-1③	-1④	Very low
	HAMD global scores	6 (398)	SMD= -0.80, 95%CI (-1.17, -0.44)	66 (p < 0.01)	-1①	-1②	0	-1③	-1④	Very low
	total clinical effectiveness rate	5 (337)	RR = 1.11, 95%CI (0.93, 1.33)	76 (p = 0.24)	-1①	-1②	0	-1③	0	Very low

Notes: GRADE [①The design of the trial has a large bias in randomization, allocation concealment, blinding or other factors; ②the confidence interval overlaps less, the heterogeneity test p is very small, the I^2 is larger, and the heterogeneity could not be completely explained by conducting subgroup analysis, sensitivity analysis, or meta-regression; ③the confidence interval is not narrow enough; ④funnel graph asymmetry]

Abbreviations: PSQI, Pittsburgh Sleep Quality Index; HAMD, Hamilton Depression Scale; SDS, Self-rating Depression Scale
